# The Bis(ferrocenyl)phosphenium Ion Revisited

**DOI:** 10.1002/anie.201913081

**Published:** 2019-12-16

**Authors:** Marian Olaru, Alexandra Mischin, Lorraine A. Malaspina, Stefan Mebs, Jens Beckmann

**Affiliations:** ^1^ Institut für Anorganische Chemie und Kristallographie Universität Bremen Leobener Straße 7 28359 Bremen Germany; ^2^ Institut für Experimentalphysik Freie Universität Berlin Arnimallee 14 14195 Berlin Germany

**Keywords:** bond analysis, carbene analogue, divalent phosphorus, ferrocene, phosphenium ions

## Abstract

The bis(ferrocenyl)phosphenium ion, [Fc_2_P]^+^, reported by Cowley et al. (*J. Am. Chem. Soc*. **1981**, *103*, 714–715), was the only claimed donor‐free divalent phosphenium ion. Our examination of the molecular and electronic structure reveals that [Fc_2_P]^+^ possesses significant intramolecular Fe⋅⋅⋅P contacts, which are predominantly electrostatic and moderate the Lewis acidity. Nonetheless, [Fc_2_P]^+^ undergoes complex formation with the Lewis bases PPh_3_ and IPr to give the donor–acceptor complexes [Fc_2_P(PPh_3_)]^+^ and [Fc_2_P(IPr)]^+^ (IPr=1,3‐bis(2,6‐diisopropylphenyl)imidazole‐2‐ylidene).

## Graphical abstract

**Lineup of suspects**: Interest in kinetically stabilized pnictogenium ions prompted the re‐investigation of the bis(ferrocenyl)phosphenium ion, the only claimed donor‐free divalent phosphenium ion. Iron was found guilty of charge compensation.

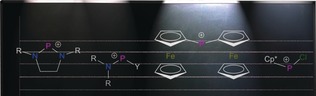

Divalent phosphenium ions, [R_2_P]^+^, are highly reactive, six‐valence‐electron species that contain an electron lone pair and a vacant p orbital.^1^ Due to the positive charge they are significantly more Lewis acidic than the isoelectronic silylenes, R_2_Si,^2^ a property also shared with the related silyl cations, [R_3_Si]^+^.^3^ Unlike those, only two substituents are available to shield the phosphorus atom and to prevent the counter anion from coordination. Consequently, the vast majority of phosphenium ions reported in the literature are electronically stabilized by substituents or ligands with donor atoms that compensate the electron deficiency, which dramatically reduces the Lewis acidity and reactivity.^1^ Although these species are unarguably cationic and many even divalent, they formally possess more than six valence electrons.^1^ These electron‐rich phosphenium ions also include the bis(supracyclopentadienyl)phosphenium ion Cp*_2_P^+^.^4^ The only notable exception seems to be the bis(ferrocenyl)phosphenium ion [Fc_2_P]^+^, reported by Cowley et al. in 1981, which allegedly lacks any donor atoms.^5^ On the basis of ^57^Fe Mössbauer and ^31^P NMR spectroscopy it was concluded that the positive charge was formally situated at phosphorus and that the iron atoms are in the oxidation state II. The claim was further supported by the ability of [Fc_2_P]^+^ to react as a Lewis acid towards the Lewis base *n*‐Bu_3_P, giving rise to the donor–acceptor complex [Fc_2_P(P*n*‐Bu_3_)]^+^.^6^ Unfortunately, neither [Fc_2_P]^+^ nor [Fc_2_P(P*n*‐Bu_3_)]^+^ have been fully characterized.^5^, ^6^ Our interest in kinetically stabilized phosphenium ions and their heavier Group 15 analogues^7^ prompted us to investigate the molecular and electronic structure of [Fc_2_P]^+^ and two related donor–acceptor complexes.

The reaction of FcLi^8^ with *i*‐Pr_2_NPCl_2_ and the subsequent treatment with water‐free HCl provided Fc_2_PCl (**1**) as yellow crystals in 63 % yield (Scheme 1).^9^ Chloride abstraction from Fc_2_PCl (**1**) was achieved using NaBAr^F^_4_ (Ar^F^=3,5‐(F_3_C)_2_C_6_H_3_), which produced [Fc_2_P][BAr^F^_4_] (**2**) as dark brown (almost black) crystals in 87 % yield (Scheme 1).^10^ The reaction of **2** with PPh_3_ and 1,3‐bis(2,6‐diisopropylphenyl)imidazole‐2‐ylidene (IPr) gave rise to the donor–acceptor complexes [Fc_2_P(PPh_3_)][BAr^F^_4_] (**3**) and [Fc_2_P(IPr)][BAr^F^_4_] (**4**), respectively, which were isolated as orange crystals in 86 % and 83 % yield.

**Scheme 1:**
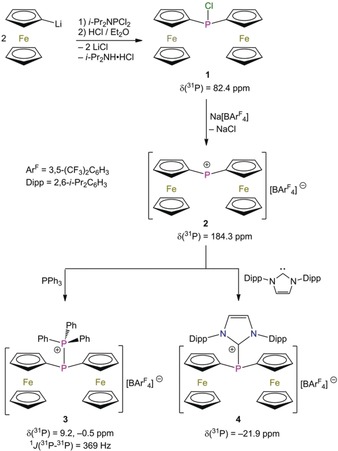
Synthesis and ^31^P NMR (CD_2_Cl_2_) chemical shifts of **1**–**4**.

Unlike Fc_2_PCl (**1**), the borate salts [Fc_2_P][BAr^F^_4_] (**2**), [Fc_2_P(PPh_3_)][BAr^F^_4_] (**3**), and [Fc_2_P(IPr)][BAr^F^_4_] (**4**) show clear ion separation in the crystal lattice (Figure 1).^11^ The most striking features of **2** are the intramolecular Fe⋅⋅⋅P contacts and the associated distortion of the molecular structure of the [Fc_2_P]^+^ cation, which are absent in **1**, **3**, and **4**. The molecular structure of [Fc_2_P]^+^ is strongly asymmetric and displays two distinctively different P‐C bonds.

**Figure 1:**
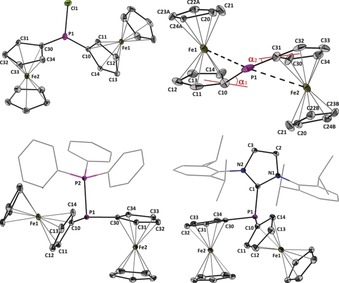
Molecular structures of Fc_2_PCl (**1**) and the cations [Fc_2_P]^+^ (**2**), [Fc_2_P(PPh_3_)]^+^ (**3**), and [Fc_2_P(IPr)]^+^ (**4**) showing 30 % probability ellipsoids. The hydrogen atoms and counter ions are omitted for clarity. Selected bond lengths [Å] and angles [°]: for **1**: P1‐C10 1.799(2), P1‐C30 1.805(2), P1‐Cl1 2.0984(7), Cl1‐P1‐C10 99.685, Cl1‐P1‐C30 99.72(5), C10‐P1‐C30 99.92(7); for **2**: Fe1‐P1 2.613(1), Fe2‐P1 3.062(1), P1‐C10 1.801(5), P1‐C31 1.714(6), C10‐P1‐C31 107.8(3), α_1_ 36.5, α_2_ 21.8; for **3**: P1‐P2 2.2335(5), P1‐C10 1.805(1), P1‐C30 1.810(1), P2‐P1‐C10 106.09(4), P2‐P1‐C30 95.86(4), C10‐P1‐C30 103.50(6); for **4**: P1‐C1 1.865(2), P1‐C10 1.818(2), P1‐C30 1.802(2), C1‐P1‐C10 96.08(9), C1‐P1‐C30 103.50(9), C10‐P1‐C30 104.04(9).

The P1‐C10 bond length in **2** (1.801(5) Å) is comparable to the related P‐C bond lengths of **1** (1.799(2) and 1.805(2) Å), **3** (1.805(1) and 1.810(1) Å), and **4** (1.818(2) and 1.802(2) Å) while the P1‐C31 bond (1.714(6) Å) is significantly shorter, by almost 0.1 Å. The latter value is closer to a P‐C double bond than a P‐C single bond, which to some degree allows the interpretation of **2** as being a phosphafulvenyl cation rather than a phosphenium cation. This view is further supported by the rather large ^1^ *J*(^31^P–^13^C) coupling of 57 Hz.^12^ The difference in the P‐C bond lengths seems to be reversely correlated with the Fe–P distances; that is, Fe1‐P1 (2.613(1) Å) is considerably shorter than Fe2‐P1 (3.062(1) Å). The two different dip angles, α_1_ (36.5°) and α_2_ (21.8°), defined as the angles made by the P‐C bond vectors with the Cp plane, reflect the same trend (Figure 1). The dip angles in **2**, although smaller than that observed for [Fc(*t*‐Bu)MeSi]^+^ (44.8°) remain noticeably larger than those observed for some Lewis acidic ferrocenylboranes such as FcBBr_2_ (18.9°, 17.7°) and FcB(C_6_F_5_)_2_ (16°), or the carbenium ion [FcCPh_2_]^+^ (20.7°).^13^ The C10‐P1‐C31 angle in **2** (107.8(3)°) is wider than the related C‐P‐C angles in **1** (99.72(5)°), **3** (103.50(6)°), and **4** (104.04(9)°). In **2**, the tilt angles between the Cp rings in Fc1 (containing Fe1, 13.3°) and Fc2 (containing Fe2, 12.3°) are similar to that observed for [Fc(*t*‐Bu)MeSi]^+^ (11.6°) and represent a significant deviation from the expected situation in **1**, where the Cp rings of the two Fc units are essentially parallel. For **3** and **4** only one of the Fc units shows a significant tilt angle, albeit of lesser value (5.2° in 3; 4.7° in **4**). In the donor–acceptor complexes **3** and **4**, the P1‐P2 (2.2335(5) Å) and P1‐C1 (1.865(2) Å) bond distances compare well with those found in the related compounds [Ph_2_P(PPh_3_)][GaCl_4_]^14^ (2.220(6) Å) and [Ph_2_P(SIMes)][B(C_6_F_5_)_4_] (1.861(4) Å, SIMes=1,3‐dimesitylimidazolidin‐2‐ylidene).^15^ No other major structural features were observed around the [Fc_2_P]^+^ fragments in these complexes, which indicates that the ligands effectively compensate most of the positive charge on P1. This was also evident by NMR spectroscopy. The formation of **2** was confirmed by the observation at a high chemical shift of its ^31^P NMR resonance signal at 184.3 ppm^10^ (CD_2_Cl_2_), shifted by more than 100 ppm compared to **1** (CD_2_Cl_2_, 82.4 ppm). In solution, the ferrocenyl groups of **2** are magnetically equivalent. Upon coordination of the Lewis base, the ^31^P{^1^H} resonance signal assigned to the [Fc_2_P]^+^ fragment shifted to −0.5 ppm in the case of **3** (d, ^1^ *J*(^31^P–^31^P)=369 Hz) and −21.9 ppm in the case of **4**.

In the gas phase the optimized molecular structure of **2** is nearly symmetric, with the two P‐C bonds of 1.75 Å and two intramolecular P‐Fe distances of 2.78 Å. For a more detailed analysis of the bonding situation a comprehensive analysis was conducted including the real‐space bonding indicators (RSBI) based upon the atoms in molecules (AIM),^16^ noncovalent interaction (NCI) index,^17^ and the electron localizability indicator (ELI‐D) methods^18^ (see Figure 2 and Tables S3–S5). The AIM bond topological analysis of the electron density (ED) shows bond paths (and thus bond critical points, bcp) for all primary P‐C, C‐C, and C‐H interactions as well as for one secondary H⋅⋅⋅H contact between the two ferrocenyl groups (Figure 2 a). However, it does not show all 20 conceivable Fe‐C bond paths, which is a common feature in AIM and related to the flat ED gradient in the conical Fe–C_5_H_5_ area.^19^ This may also explain why there is no bcp formed between the P atom and the two Fe atoms. To get a more detailed view into that, the ED distribution was mapped on the surface of the AIM atomic basin of the P atom, which discloses strong ED accumulations along the two P‐C axes but no apparent accumulation in the P⋅⋅⋅Fe area, suggesting this interaction to be noncovalent (Figure 2 b). The NCI complements AIM in that it uncovers regions in space where (weak) noncovalent interactions occur even if no AIM bond paths were observed.^20^ Accordingly, ring‐shaped and red‐colored NCI basins are obtained for every Fe–C_5_H_5_ contact, suggesting dominant covalent metal to cyclopentadienyl interactions, as well as small green areas corresponding to the weak H⋅⋅⋅H contacts (Figure 2 c). Notably, localized and blue‐colored NCI areas are obtained along the P‐Fe axis, indicating an (almost) purely noncovalent atom–atom contact. This is supported by the ELI‐D, which does not show any P‐Fe bonding basins (Figure 2 d). To investigate whether regions of increased electron localizability are formed between the P and the Fe atoms, the ELI‐D distribution was mapped on the lone‐pair basins of the P atom (Figure 2 e) as well as the adjacent P‐C bonding basin (Figure 2 f). Although the lone‐pair basin of the P atom shows a small excrescence in direction of the Fe atom, no indications for increased electron localizability are present for both basin types, supporting the weak noncovalent nature of the P⋅⋅⋅Fe contact. A quantitative measure is given by the Raub–Jansen Index (RJI),^21^ which overlaps ELI‐D basins with AIM basins and proves that only 0.05 e of the P atoms ELI‐D lone‐pair basin are located within each AIM atomic basin of the two Fe atoms.

**Figure 2:**
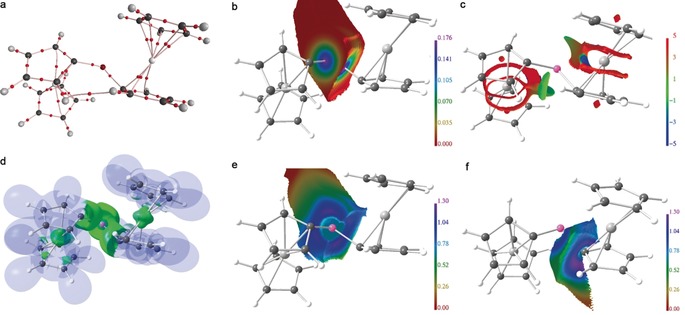
Real‐space bond indicator (RSBI) analysis of the cations [Fc_2_P]^+^ (**2**). a) AIM bond topological analysis, b) ED distribution mapped on the surface of the AIM atomic basin of the P atom, c) NCI basins indicating noncovalent interactions, d) ELI‐D distribution indicating regions of electron localizability, e) ELI‐D distribution mapped on the lone‐pair basin of the P atom, and f) the adjacent P‐C bonding basin.

Complementary to the ED based methods, molecular orbital (MO) and natural bond orbital (NBO) calculations of **2** were carried out. The HOMOs contain Fe(d_xy_, d${}_{x{}^{2}-y{}^{2}}$ ), P(s,p_x_,p_y_), and C(s,p) contributions, which are responsible for the Fe‐C and P‐C interactions. The LUMO is given by the empty p_z_ orbital of the P atom, whereas higher LUMOs are given by antibonding Fe‐C contributions (see the Supporting Information). The Wiberg bond index (WBI) of the P‐C interactions is 1.04, thus excluding hyperconjugation. The P‐C NBOs are populated with 1.96 e, 67 % of which stems from the C atoms’ atomic orbitals. p‐Type contributions are 66 % for the sp^2^‐C atoms and 81 % for the P atoms confirming WBI. The WBIs of the P⋅⋅⋅Fe contacts are as small as 0.152, emphasizing once again the low covalent bond character.^22^ The elusive phosphenium ions [Me_2_P]^+^ and [Ph_2_P]^+^ were predicted to be Lewis superacids in the gas phase.^23^ We finally calculated the fluoride ion affinity (FIA) of **2** (670 kJ mol^−1^), which is substantially smaller than that of [Me_2_P]^+^ (935 kJ mol^−1^), and only slightly smaller than that of [Ph_2_P]^+^ (789 kJ mol^−1^). Yet the value is still larger than that of SbF_5_ (480 kJ mol^−1^), which classifies [Fc_2_P]^+^ (**2**) also as a Lewis super acid.^24^

In summary, we investigated the molecular and electronic structure of the phosphenium ion [Fc_2_P]^+^ (**2**) first reported by Cowley et al. in 1981^5^, ^6^ Despite their previous assumption, it possesses significant intramolecular Fe⋅⋅⋅P contacts, which distort the ideal geometry and increase the coordination number at the P atom. These Fe⋅⋅⋅P contacts are predominantly electrostatic and moderate the Lewis acidity. Nonetheless, [Fc_2_P]^+^ (**2**) is still a Lewis superacid and undergoes complexation with typical Lewis bases, such as PPh_3_ and IPr, to give the donor–acceptor complexes [Fc_2_P(PPh_3_)]^+^ (**3**) and [Fc_2_P(IPr)]^+^ (**4**). We are currently studying the utility of **2** for the activation of small molecules.

## Conflict of interest

The authors declare no conflict of interest.

## Supporting information

As a service to our authors and readers, this journal provides supporting information supplied by the authors. Such materials are peer reviewed and may be re‐organized for online delivery, but are not copy‐edited or typeset. Technical support issues arising from supporting information (other than missing files) should be addressed to the authors.

Supplementary
