# Supplementary material for: The Bis(ferrocenyl)phosphenium Ion Revisited
Source: Angew Chem Int Ed Engl. 2019 Dec 16;59(4):1581–4. doi: 10.1002/anie.201913081 (PMC7003730; doi:10.1002/anie.201913081)
Supplement: Supplementary file 1 — Supplementary [file ANIE-59-1581-s001.pdf]

Angewandte Chemie
Eine Zeitschrift der Gesellschaft Deutscher Chemiker

Supporting Information

The Bis(ferrocenyl)phosphenium Ion Revisited

Marian Olaru, Alexandra Mischin, Lorraine A. Malaspina, Stefan Mebs, and Jens Beckmann**

anie_201913081_sm_miscellaneous_information.pdf

Table of Contents

Experimental procedures 2

General information 2

Synthesis and characterization of Fc_2PCl (**1**) 3

Synthesis and characterization of $[Fc_2P][BAr^F_4]$ (**2**) 6

Synthesis and characterization of $[Fc_2P(PPh_3)][BAr^F_4]$ (**3**) 10

Synthesis and characterization of $[Fc_2P(IPr)][BAr^F_4]$ (**4**) 14

X-Ray diffraction studies 18

Computational data 21

References 33

Experimental procedures

General information

All reactions, manipulations, work-up and purifications were performed under inert argon atmosphere using anhydrous solvents. Unless otherwise stated, reagents used in this work including ferrocene and PCl_3 were obtained commercially and were used as received. FcLi,[1,2] Na[BAr^F_4][3,4] [Ar^F = 3,5-$(F_3C)_2C_6H_3$], *i*-Pr_2NPCl_2,[5] IPr (1,3-Bis(2,6-di-*i*-propylphenyl)imidazol-2-ylidene)[6,7] were prepared following the published procedures. Anhydrous dichloromethane, hexane, tetrahydrofuran and toluene were collected from an SPS800 mBraun solvent purification system and stored over 4 Å molecular sieves. 1,2-Difluorobenzene was degassed and dried under argon over 4 Å molecular sieves. Et_2O was dried by refluxing it over Na/benzophenone under argon atmosphere. Deuterated solvents were degassed and dried over 4 Å molecular sieves under argon.

Unless otherwise noted, NMR spectra were recorded at room temperature on a Bruker Avance Neo 600 MHz spectrometer. ^{1}H, $^{13}C\{^{1}H\}$, $^{11}B\{^{1}H\}$, $^{31}P\{^{1}H\}$ and ^{19}F NMR spectra are reported on the δ scale (ppm) and are referenced against $SiMe_4$, $BF_3{\cdot}Et_2O$ (15% in $CDCl_3$), H_3PO_4 (85% in water) and $CFCl_3$, respectively. ^{1}H and $^{13}C\{^{1}H\}$ chemical shifts are reported relative to the residual peak of the solvent ($CDHCl_2$: 5.32 ppm, for CD_2Cl_2) in the ^{1}H NMR spectra, and to the peak of the deuterated solvent (CD_2Cl_2: 53.84 ppm) in the $^{13}C\{^{1}H\}$ NMR spectra.[8] The assignment of the ^{1}H and $^{13}C\{^{1}H\}$ resonance signals was made in accordance with the COSY, HSQC and HMBC spectra.

The ESI HRMS spectra were measured on a Bruker Impact II spectrometer. Acetonitrile or dichloromethane/acetonitrile solutions ($c = 1{\cdot}10^{-5}$ mol·L^{-1}) were injected directly into the spectrometer at a flow rate of 3 µL·min^{-1}. Nitrogen was used both as a drying gas and for nebulization with flow rates of approximately 5 L·min^{-1} and a pressure of 5 psi. Pressure in the mass analyzer region was usually about $1{\cdot}10^{-5}$ mbar. Spectra were collected for 1 min and averaged. The nozzle-skimmer voltage was adjusted individually for each measurement.

Synthesis and characterization of Fc_2PCl (1)

To a pre-cooled (−80 °C) suspension of FcLi (10.77 g, 56 mmol) in THF (40 mL), *i*-Pr_2NPCl_2 (5.67 g, 28 mmol) was added. The reaction mixture was allowed to warm up to room temperature over the course of 12 h. A solution of HCl in Et_2O (1.7 M, 20 mL, 34 mmol) was added slowly. The suspension was filtered over a pad of dry Celite to remove the insoluble *i*-Pr_2NH_2Cl. After the solvents were evaporated to dryness, CH_2Cl_2 (100 mL) was added and the suspension filtered over a pad of dry Celite to remove LiCl. All volatiles were evaporated to dryness and the remaining solid was washed with MeCN (3×25 mL) at rt and Et_2O at 0°C (3×25mL). The remaining solid was dried under vacuum overnight. The title product was obtained as a yellow solid (7.72 g, 63%). **Mp**. 186–188 °C.

^{1}H NMR (600 MHz, CD_2Cl_2): δ = 4.51 (s, br, 2H, P-Cp Hα), 4.49 (s, br, 2H, P-Cp Hβ), 4.41 (s, br, 2H, P-Cp Hβ), 4.28 (s, br, 2H, P-Cp Hα), 4.20 (s, 10H, Cp). **^{13}C{^{1}H} NMR (151 MHz, CD_2Cl_2)**: δ = 79.54 (d, $^1J(^{13}C\text{-}^{31}P)$ = 29 Hz, P-Cp C_{ipso}), 73.00 (d, $^2J(^{13}C–^{31}P)$ = 20 Hz, P-Cp Cα), 72.40 (d, $^3J(^{13}C–^{31}P)$ = 4 Hz, P-Cp Cβ), 72.01 (d, $^2J(^{13}C–^{31}P)$ = 16 Hz, P-Cp Cα), 71.65 (d, $^3J(^{13}C–^{31}P)$ = 4 Hz, P-Cp Cβ), 69.91 (s, Cp). **^{31}P{^{1}H} NMR (243 MHz, CD_2Cl_2)**: δ = 82.36 (s). **HRMS ESI (m/z):** $[M−Cl+OH]^+$ calculated. for $C_{20}H_{19}Fe_2OP$, 417.98669; found, 417.98668 (hydrolysis occurred during measurement).

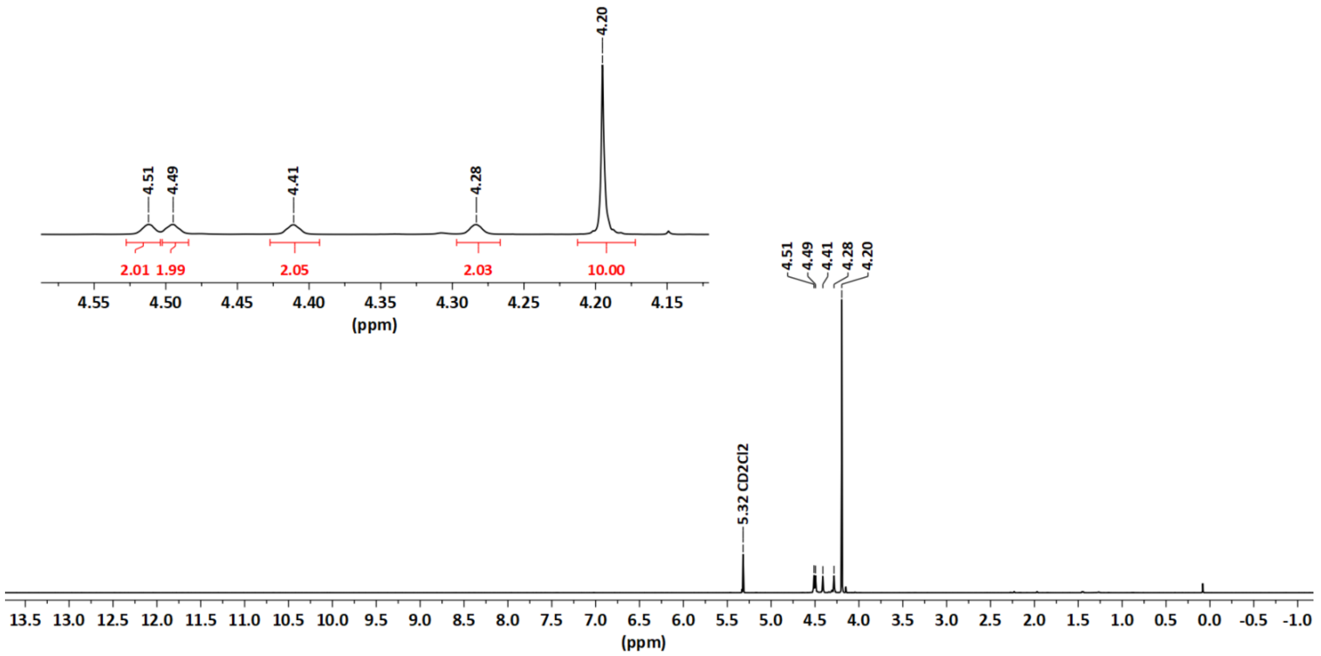

Figure S1. ^{1}H NMR (CD_2Cl_2, 600 MHz) spectrum of **1**.

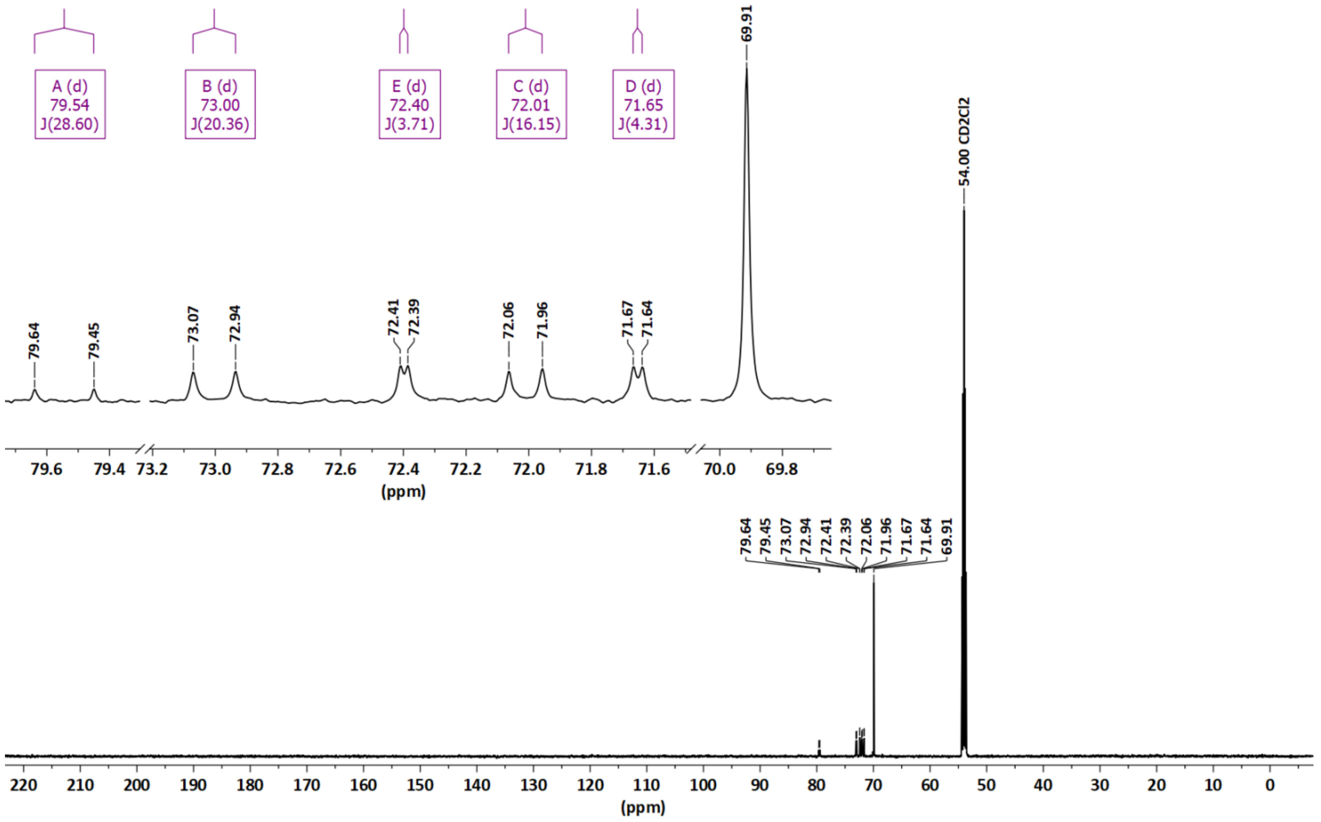

Figure S2. $^{13}C\{^{1}H\}$ NMR (CD_2Cl_2, 151 MHz) spectrum of **1**.

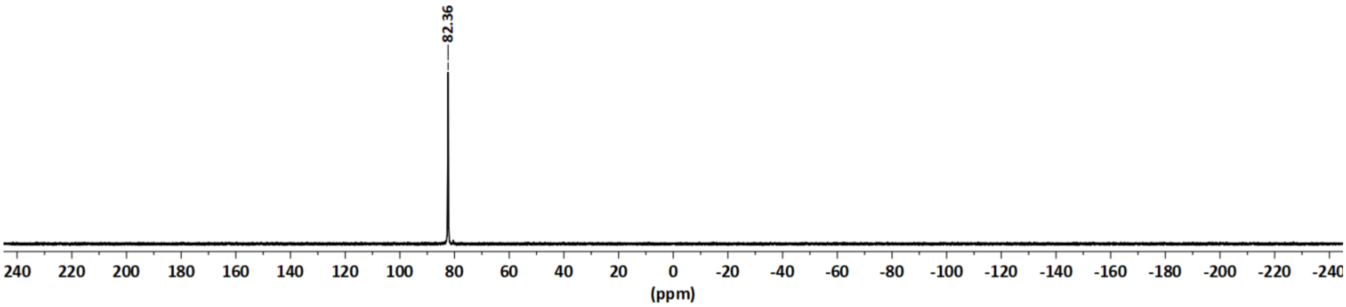

Figure S3. ^{31}P NMR (CD_2Cl_2, 243 MHz) spectrum of **1**.

Synthesis and characterization of $[Fc_2P][BAr^F_4]$ (2)

To a solid mixture of Fc_2PCl (0.437 g, 1 mmol) and $Na[BAr^F_4]$ (0.886 g, 1 mmol) was added CH_2Cl_2 (10 mL) at room temperature. The reaction mixture was stirred for 15 minutes then filtered under argon through a syringe PTFE filter; a dark red-brown solution was obtained. Hexane (50 mL) was added with vigorous stirring. The microcrystalline solid was allowed to settle, and the solution removed. The remaining solid was recrystallized once from 1,2-difluorobenzene/hexane (4 mL/20 mL) and once from CH_2Cl_2/hexane (4 mL/20 mL). The title product was obtained as a black solid (1.10 g, 87%). **Mp.** 144–146 °C. **^{1}H NMR (600 MHz, CD_2Cl_2)**: δ = 7.74 (s, br, 8H, *o*-Ar^F), 7.54 (s, 4H, *p*-Ar^F), 5.45 (m, br, 4H, P-Cp Hβ), 4.69 (s, br, 14H, P-Cp Hα + Cp). **^{13}C{^{1}H} NMR (151 MHz, CD_2Cl_2)**: δ = 162.31 (q, $^1J(^{11}B–^{13}C)$ = 50 Hz, *i*-Ar^F), 135.38 (s, *o*-Ar^F), 129.46 (qq, $^2J(^{19}F–^{13}C)$ = 32 Hz, $^4J(^{19}F–^{13}C)$ = 5 Hz, *m*-Ar^F), 125.16 (q, $^1J(^{19}F–^{13}C)$ = 273 Hz, CF_3), 118.03 (s, br, *p*-Ar^F), 92.17 (d, $^1J(^{13}C–^{31}P)$ = 57 Hz, P-Cp C_{ipso}), 83.76 (s, P-Cp Cβ), 78.52 (d, $^2J(^{13}C–^{31}P)$ = 17.92 Hz, P-Cp Cα), 75.96 (s, Cp). **^{31}P{^{1}H} NMR (243 MHz, CD_2Cl_2)**: δ = 184.31 (s), **^{19}F NMR (565 MHz, CD_2Cl_2)**: δ = −62.77 (s). **^{11}B{^{1}H} NMR (193 MHz, CD_2Cl_2)**: δ = −6.59 (s).

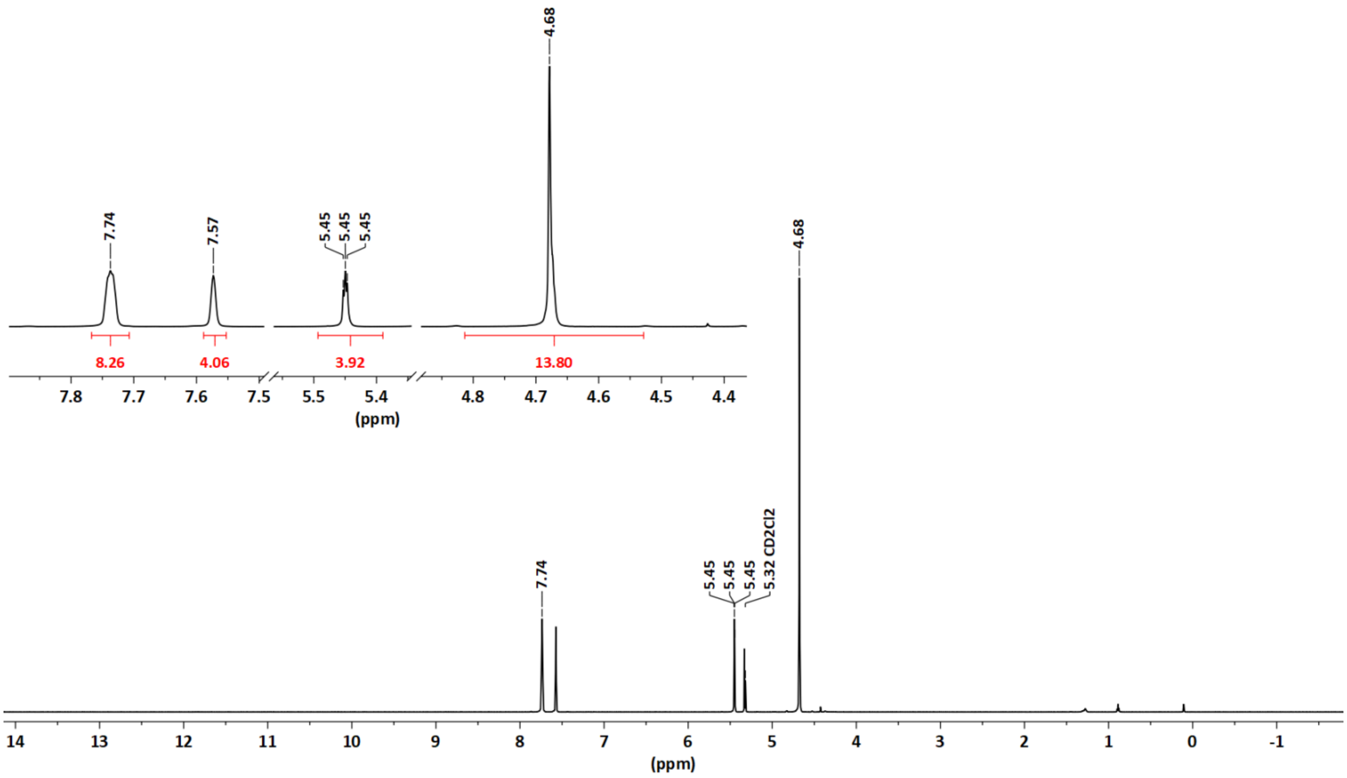

Figure S4. ^{1}H NMR (CD_2Cl_2, 600 MHz) spectrum of **2**.

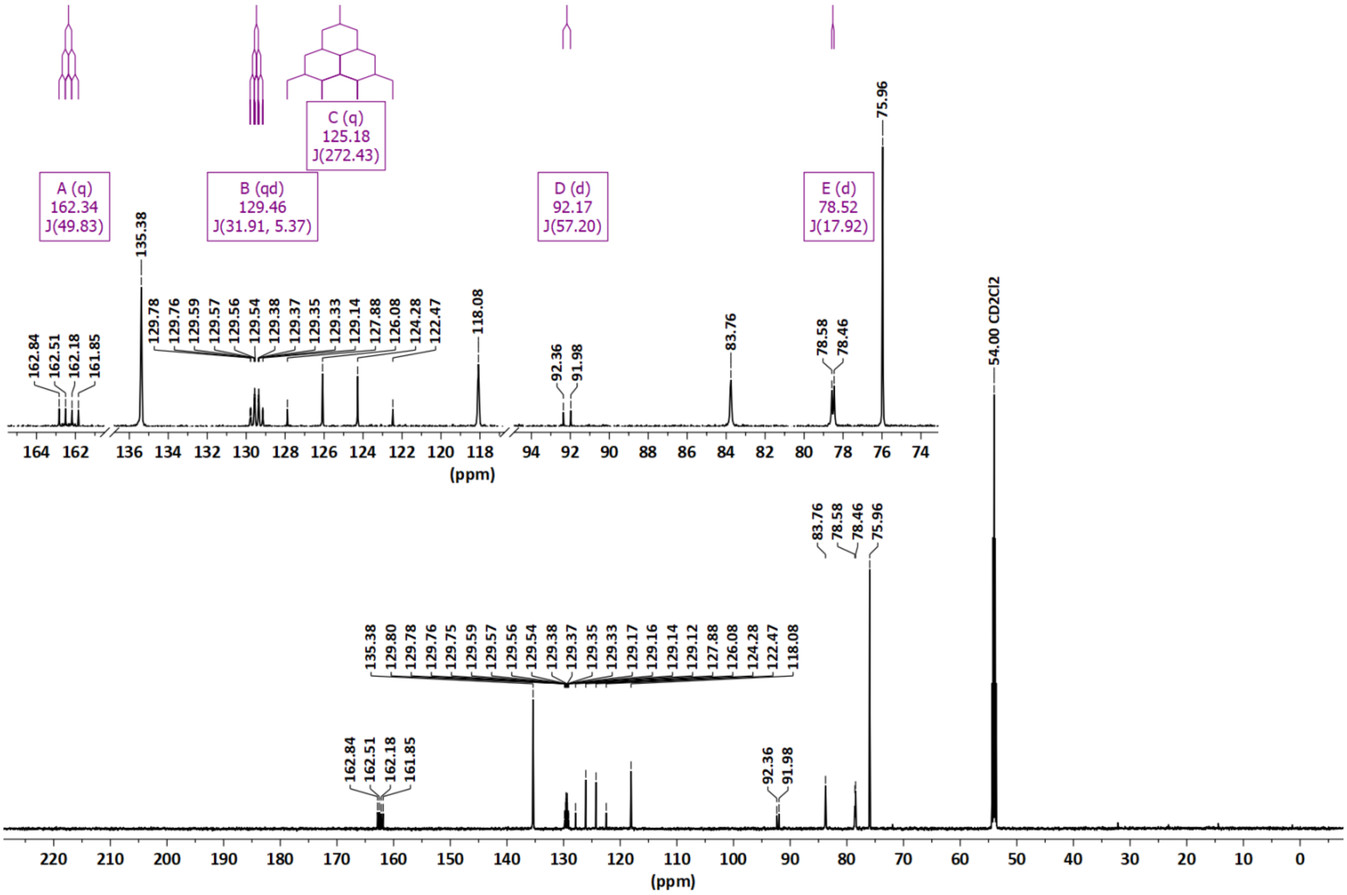

Figure S5. $^{13}C\{^{1}H\}$ NMR (CD_2Cl_2, 151 MHz) spectrum of **2**.

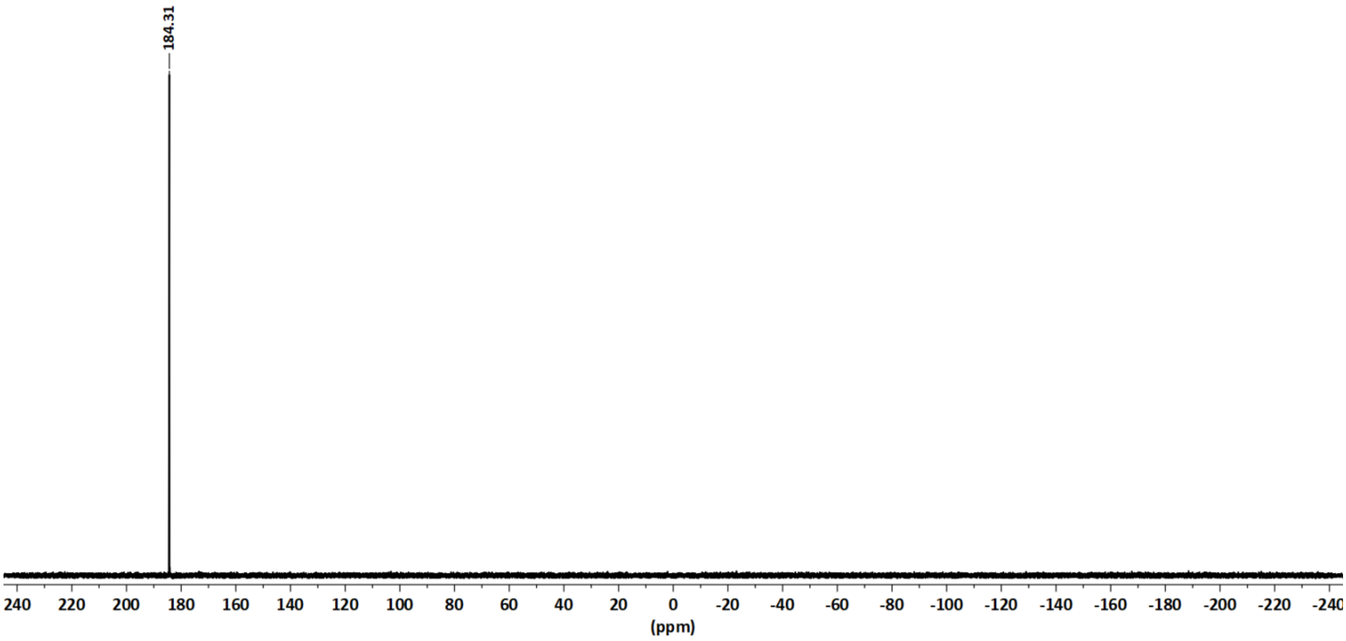

Figure S6. $^{31}P\{^{1}H\}$ NMR (CD_2Cl_2, 243 MHz) spectrum of **2**.

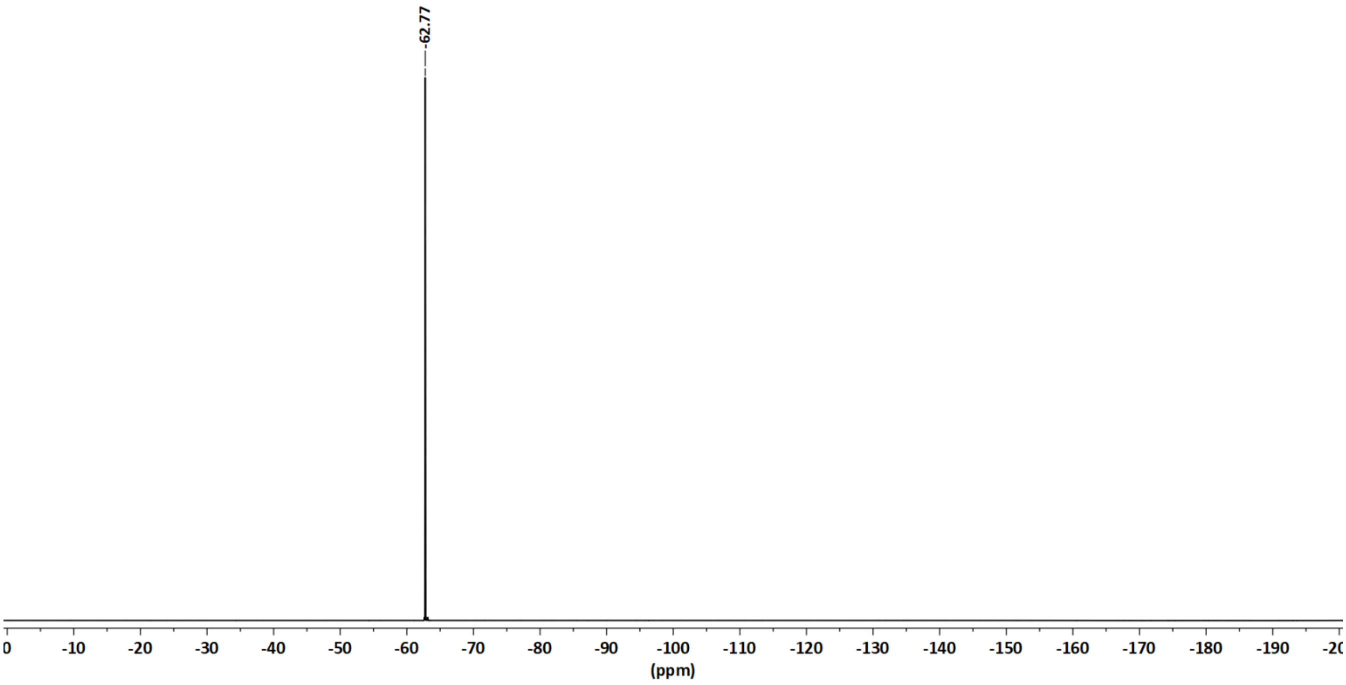

Figure S7. ^{19}F NMR (CD_2Cl_2, 565 MHz) spectrum of **2**.

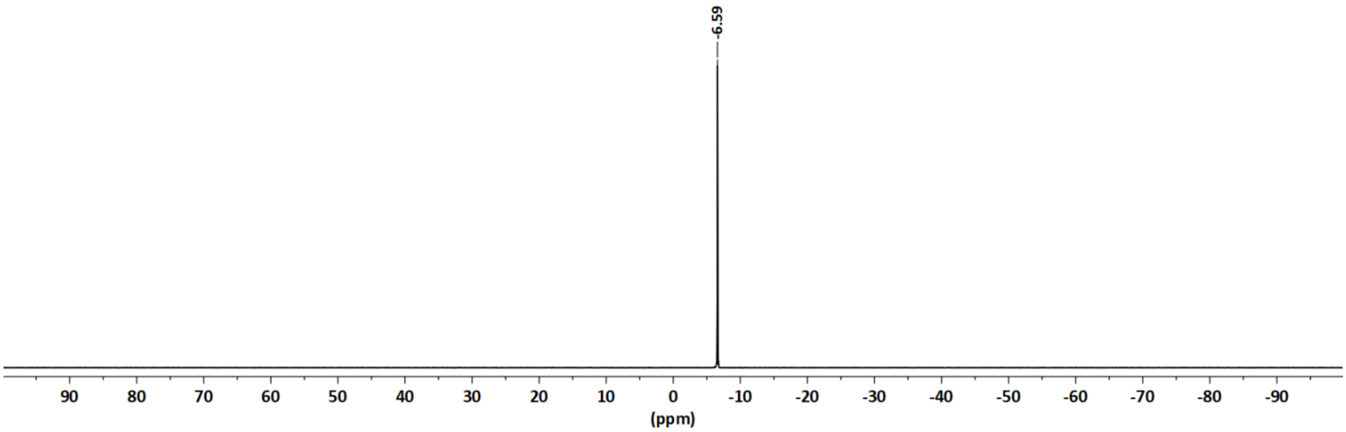

Figure S8. $^{11}B\{^1H\}$ NMR (CD_2Cl_2, 193 MHz) spectrum of **2**.

Synthesis and characterization of [$Fc_2P(PPh_3)$][BAr^F_4] (3)

To a solid mixture of Fc_2PCl (0.100 g, 0.23 mmol), Na[BAr^F_4] (0.203 g, 0.23 mmol) and PPh_3 (61 mg, 0.23 mmol) was added CH_2Cl_2 (5 mL). The orange reaction mixture was stirred for 15 min at room temperature and afterwards filtered through a PTFE syringe filter. Hexane (25 mL) was layered. After 48 h the crystalline product was isolated and dried at reduced pressure. The title compound was obtained as an orange solid (0.131 g, 86 %). **Mp.** 170–172 °C. **^{1}H NMR (600 MHz, CD_2Cl_2)**: δ = 7.73 (m, 11H, *o*-Ar^F + *p*-Ph), 7.55 (s, 4H, *p*-Ar^F), 7.54 (m, 6H, *m*-Ph), 7.30 (m, 6H, *o*-Ph), 4.59 (m, br, 2H, P-Cp Hβ), 4.46 (m, br, 2H, P-Cp Hα), 4.44 (m, br, 2H, P-Cp Hβ), 4.37 (s, 10H, Cp), 3.81 (m, br, 2H, P-Cp Hα). **^{13}C{^{1}H} NMR (151 MHz, CD_2Cl_2)**: δ = 162.29 (m, *i*-Ar^F), 135.32 (s, *o*-Ar^F), 135.15 (d, $^4J(^{13}C–^{31}P)$ = 3 Hz, *p*-Ph), 134.67 (dd, $^2J(^{13}C–^{31}P)$ = 9 Hz, $^4J(^{13}C–^{31}P)$ = 3 Hz, *o*-Ph) 130.56 (d, $^3J(^{13}C–^{31}P)$ = 12 Hz, *m*-Ph) 129.44 (qq, $^2J(^{19}F–^{13}C)$ = 31 Hz, $^4J(^{19}F–^{13}C)$ = 3 Hz, *m*-Ar^F), 125.13 (q, $^1J(^{19}F–^{13}C)$ = 272 Hz, CF_3), 119.77 (dd, $^1J(^{13}C–^{31}P)$ = 65 Hz, $^2J(^{13}C–^{31}P)$ = 6 Hz, *i*-Ph), 118.02 (quintet, br, $^3J(^{19}F–^{13}C)$ = 4 Hz, *p*-Ar^F), 74.80 (dd, $^2J(^{13}C–^{31}P)$ = 27 Hz, $^3J(^{13}C–^{31}P)$ = 2 Hz, P-Cp Cα), 73.44 (d, $^3J(^{13}C–^{31}P)$ = 3 Hz, P-Cp Cβ), 73.14 (s, P-Cp Cβ), 72.99 (d, $^2J(^{13}C–^{31}P)$ = 7 Hz, P-Cp Cα), 70.88 (s, Cp), 64.13 (d, $^1J(^{13}C–^{31}P)$ = 7 Hz, P-Cp C_{ipso}). **^{31}P{^{1}H} NMR (243 MHz, CD_2Cl_2)**: δ = 9.24 (d, $^1J(^{31}P–^{31}P)$ = 369 Hz, PPh_3), −0.50 (d, $^1J(^{31}P–^{31}P)$ = 369 Hz, P-Cp), **^{19}F NMR (565 MHz, CD_2Cl_2)**: δ = −62.82 (s). **^{11}B{^{1}H} NMR (193 MHz, CD_2Cl_2)**: δ = −6.59 (s). **HRMS ESI (m/z):** [Fc_2P+OH+Na]$^+$ calculated. for $C_{20}H_{19}Fe_2NaOP$, 440.97646; found, 440.97651 (hydrolysis occurred during measurement).

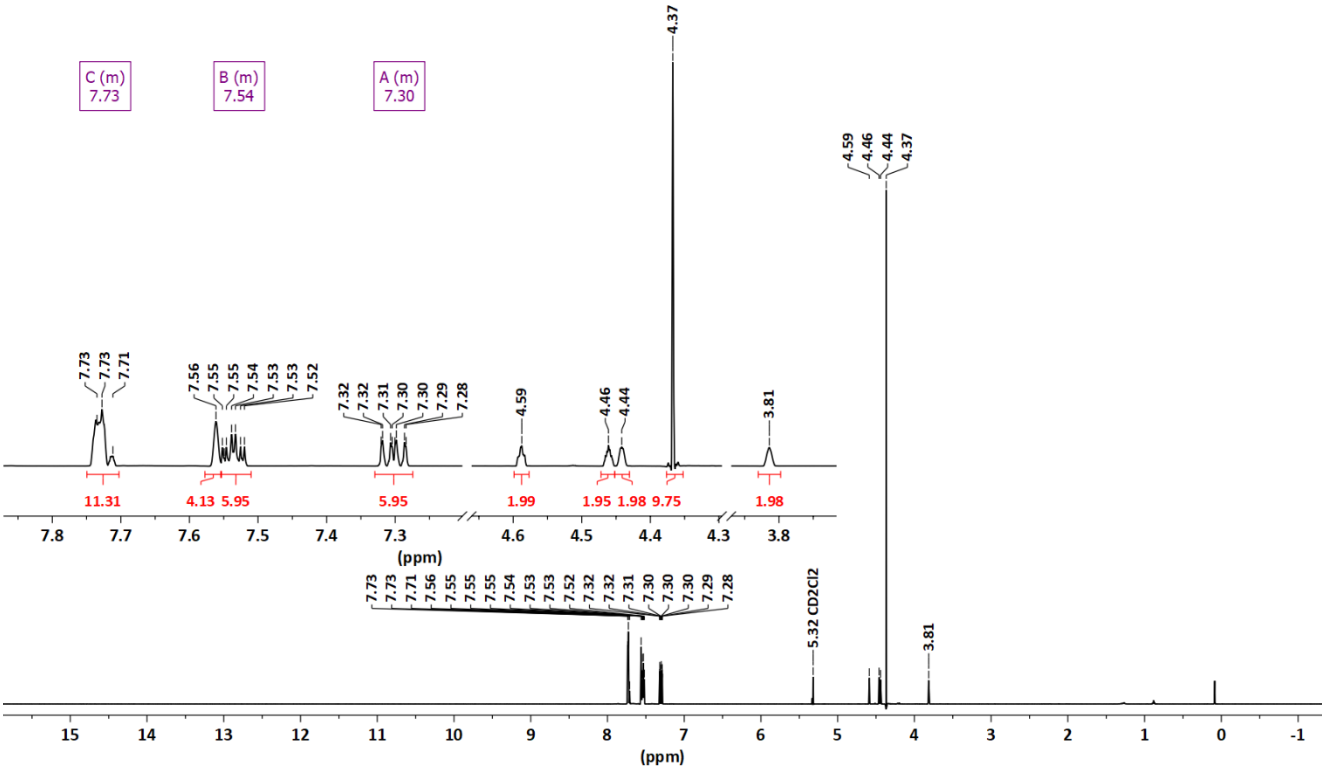

Figure S9. ^{1}H NMR (CD_2Cl_2, 600 MHz) spectrum of **3**.

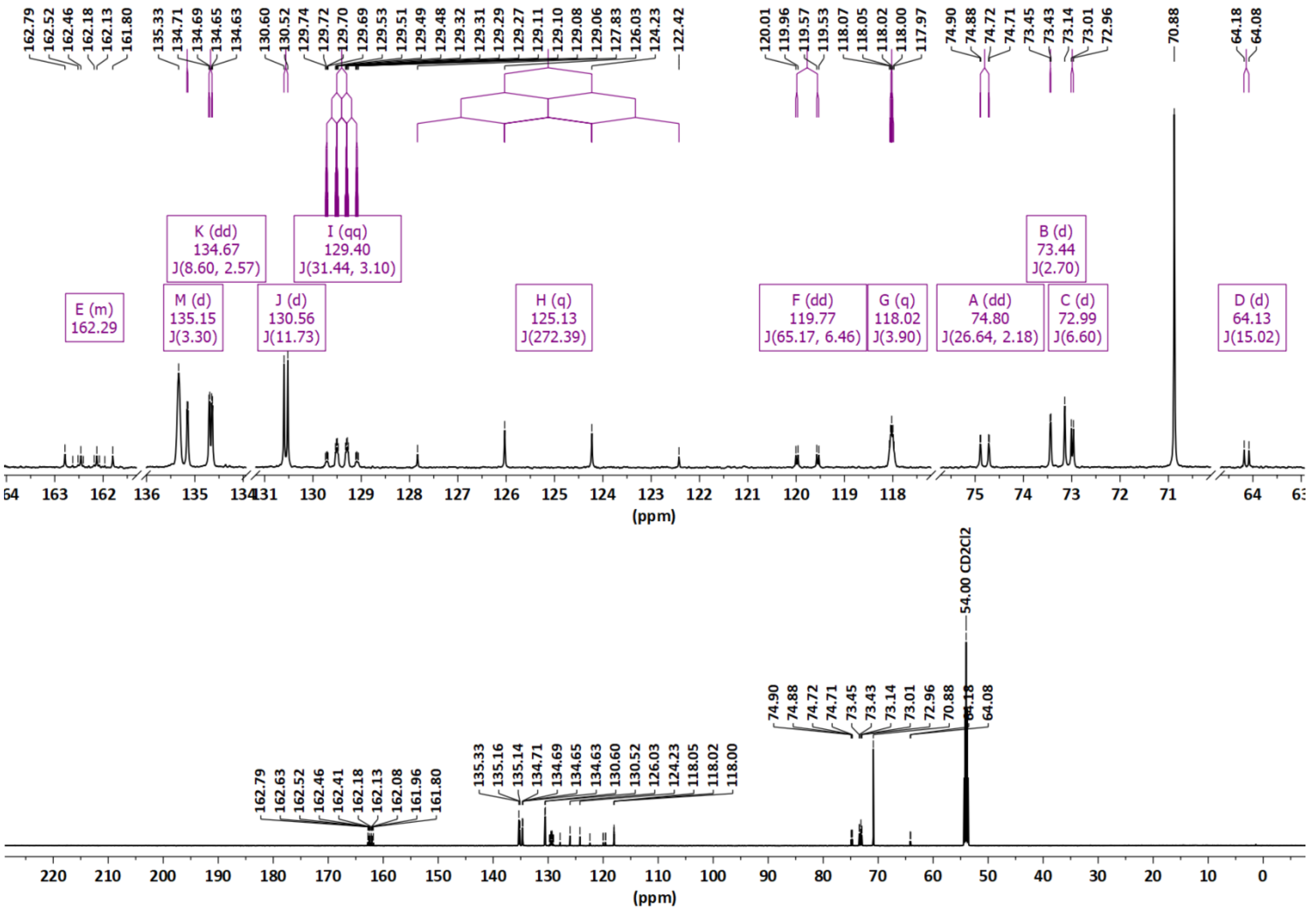

Figure S10. $^{13}C\{^{1}H\}$ NMR (CD_2Cl_2, 151 MHz) spectrum of **3**.

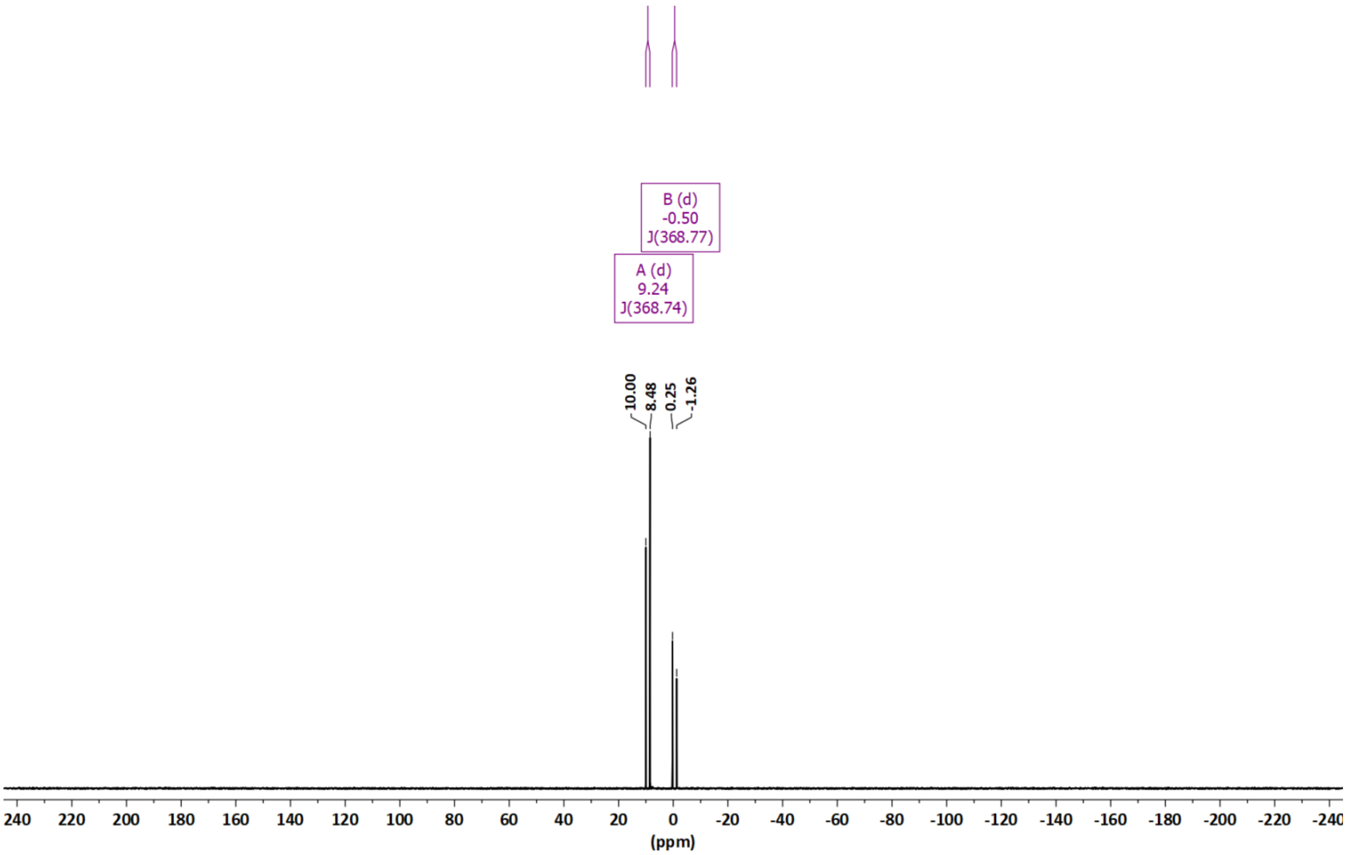

Figure S11. $^{31}P\{^{1}H\}$ NMR (CD_2Cl_2, 243 MHz) spectrum of **3**.

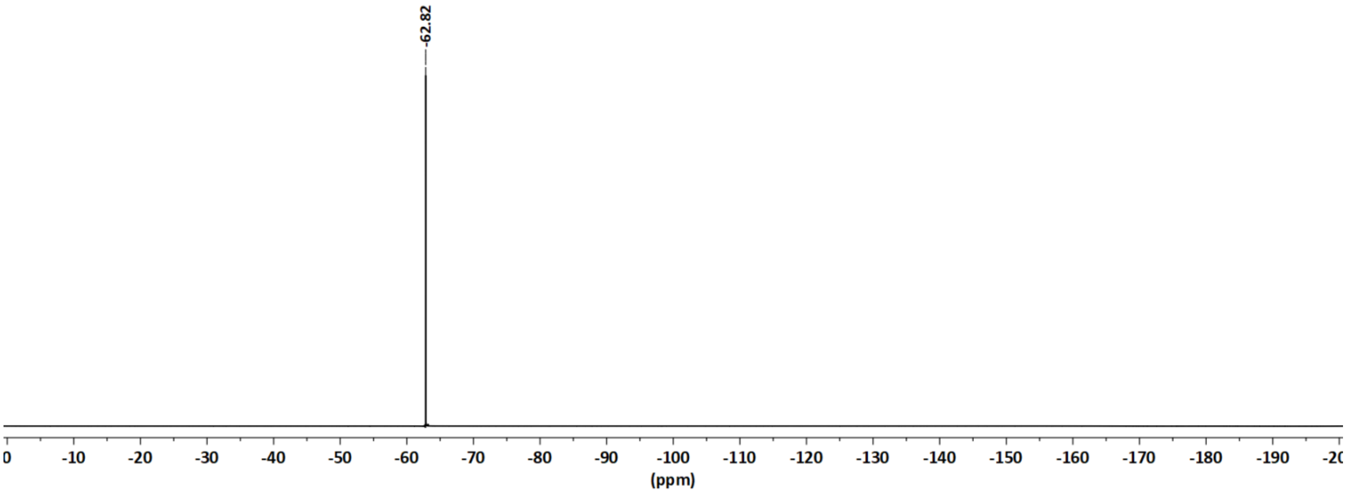

Figure S12. ^{19}F NMR (CD_2Cl_2, 565 MHz) spectrum of **3**.

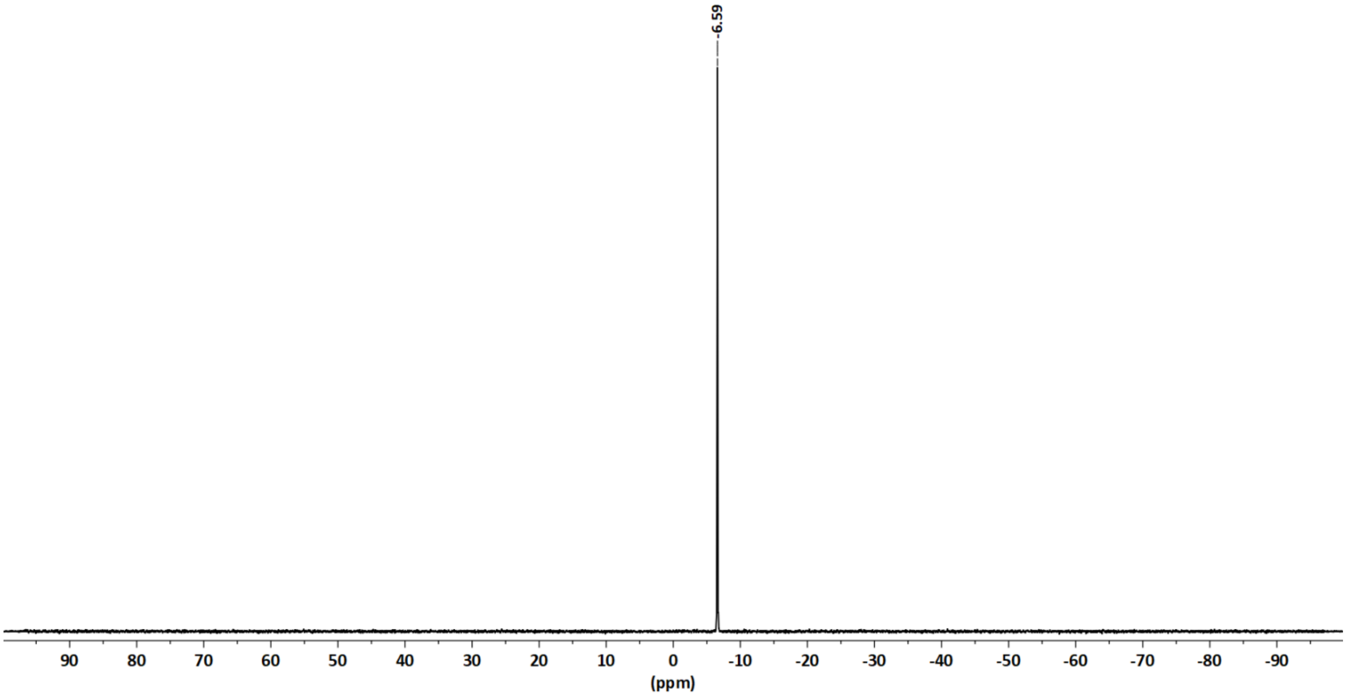

Figure S13. $^{11}B\{^{1}H\}$ NMR (CD_2Cl_2, 193 MHz) spectrum of **3**.

Synthesis and characterization of [Fc_2P(IPr)][BAr^F_4] (4)

To a solid mixture of [Fc_2P(PPh_3)][BAr^F_4] (0.275 g, 0.22 mmol) and IPr (0.085 g, 0.22 mmol) CH_2Cl_2 (15 mL) was added and the reaction mixture was stirred at room temperature for 20 minutes. Hexane (24 mL) was layered. After 48 h the crystalline product was isolated and dried at reduced pressure. The title compound was obtained as an orange solid (0.298 g, 83%). **Mp.** 196–198 °C. **^{1}H NMR (600 MHz, CD_2Cl_2)**: *δ* = 7.73 (m, 11H, *o*-Ar^F), 7.57 (s, 4H, *p*-Ar^F), 7.54 (t, br, $^3J(^1H–^1H)$ = 7 Hz, 2H, IPr *p*-Ar), 7.44 (s, 2H, IPr NC**H**), 7.31 (d, br, $^3J(^1H–^1H)$ = 7 Hz, IPr *m*-Ar), 4.43 (m, br, 2H, P-Cp Hα), 4.21 (m, br, 2H, P-Cp Hβ), 4.19 (m, br, 2H, P-Cp Hβ), 4.09 (s, 10H, Cp), 4.03 (m, br, 2H, P-Cp Hα), 4.38 (s, br, 4H, IPr H_3C-C**H**-CH_3), 1.32 (s, br, 12H, IPr **H_3**C–CH–C**H_3**), 1.07 (d, $^3J(^1H–^1H)$ = 7 Hz, 12H, IPr **H_3**C–CH–C**H_3**). **$^{13}C\{^1H\}$ NMR (151 MHz, CD_2Cl_2)**: *δ* = 162.33 (m, *i*-Ar^F), 151.17 (d, $^1J(^{31}P–^{13}C)$ = 100 Hz, IPr N**C**N), 145.16 (s, IPr *o*-Ar), 135.38 (s, *o*-Ar^F), 132.89 (s, IPr *p*-Ar), 131.71 (s, IPr *i*-Ar), 129.44 (qq, $^2J(^{19}F–^{13}C)$ = 31 Hz, $^4J(^{19}F–^{13}C)$ = 3 Hz, *m*-Ar^F), 127.37 (s, br, IPr N**C**H), 125.18 (q, $^1J(^{19}F–^{13}C)$ = 272 Hz, CF_3), 125.58 (s, IPr *m*-Ar), 118.04 (quintet, br, $^3J(^{19}F–^{13}C)$ = 4 Hz, *p*-Ar^F), 73.32 (d, $^2J(^{13}C–^{31}P)$ = 26 Hz, P-Cp Cα), 72.31 (s, P-Cp Cβ), 71.93 (d, $^2J(^{13}C–^{31}P)$ = 6 Hz, P-Cp Cα), 71.91 (s, P-Cp Cβ), 70.84 (s, Cp), 65.16 (s, P-Cp C_{ipso}), 30.22 (s, IPr H_3C–**C**H–CH_3), 26.96 (s, H_3**C**–CH–**C**H_3), 22.10 (s, H_3**C**–CH–**C**H_3). **$^{31}P\{^1H\}$ NMR (243 MHz, CD_2Cl_2)**: *δ* = −21.89 (s). **^{19}F NMR (565 MHz, CD_2Cl_2)**: *δ* = −62.82 (s). **$^{11}B\{^1H\}$ NMR (193 MHz, CD_2Cl_2)**: *δ* = −6.60 (s). **HRMS ESI (m/z):** $[M+Na]^+$ calculated. for $C_{47}H_{54}Fe_2N_2P$, 789.27179; found, 789.27159.

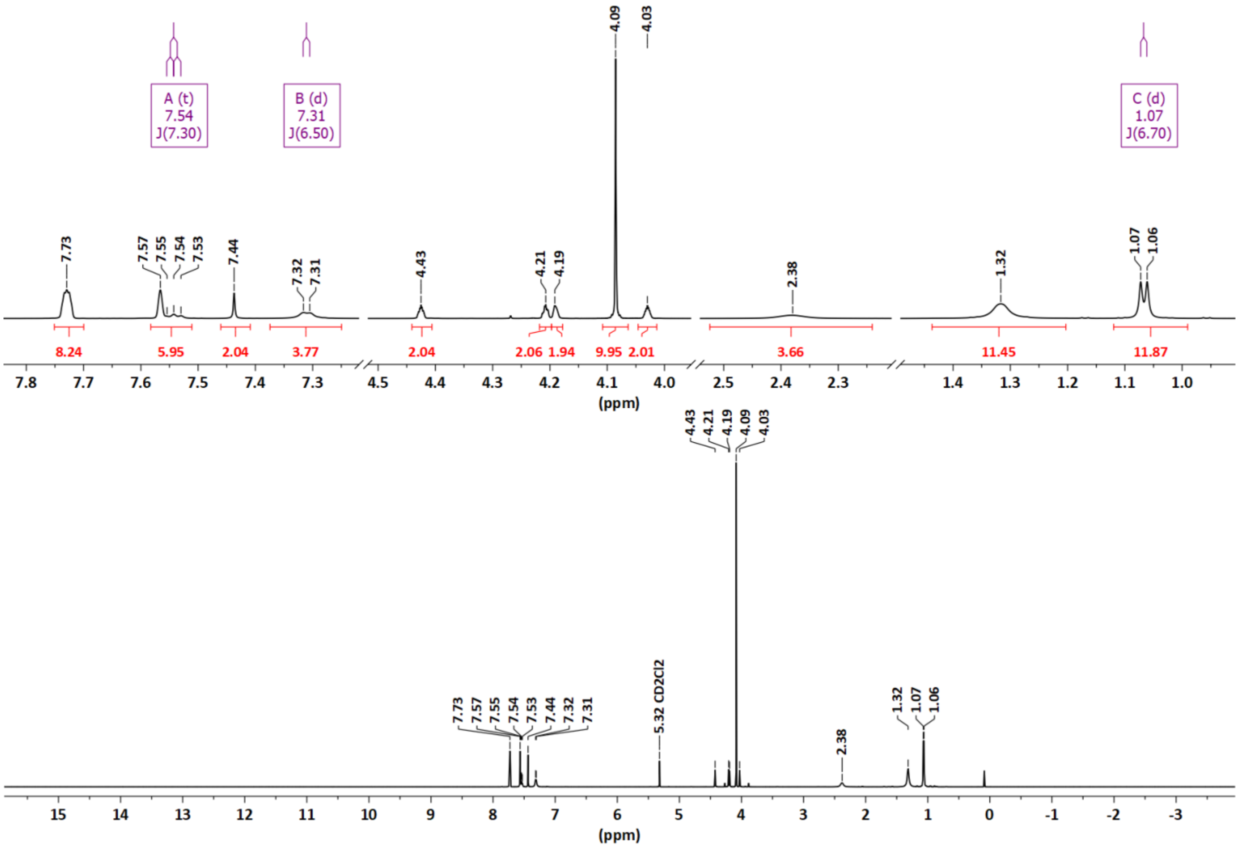

Figure S14. ^{1}H NMR (CD_2Cl_2, 600 MHz) spectrum of **4**.

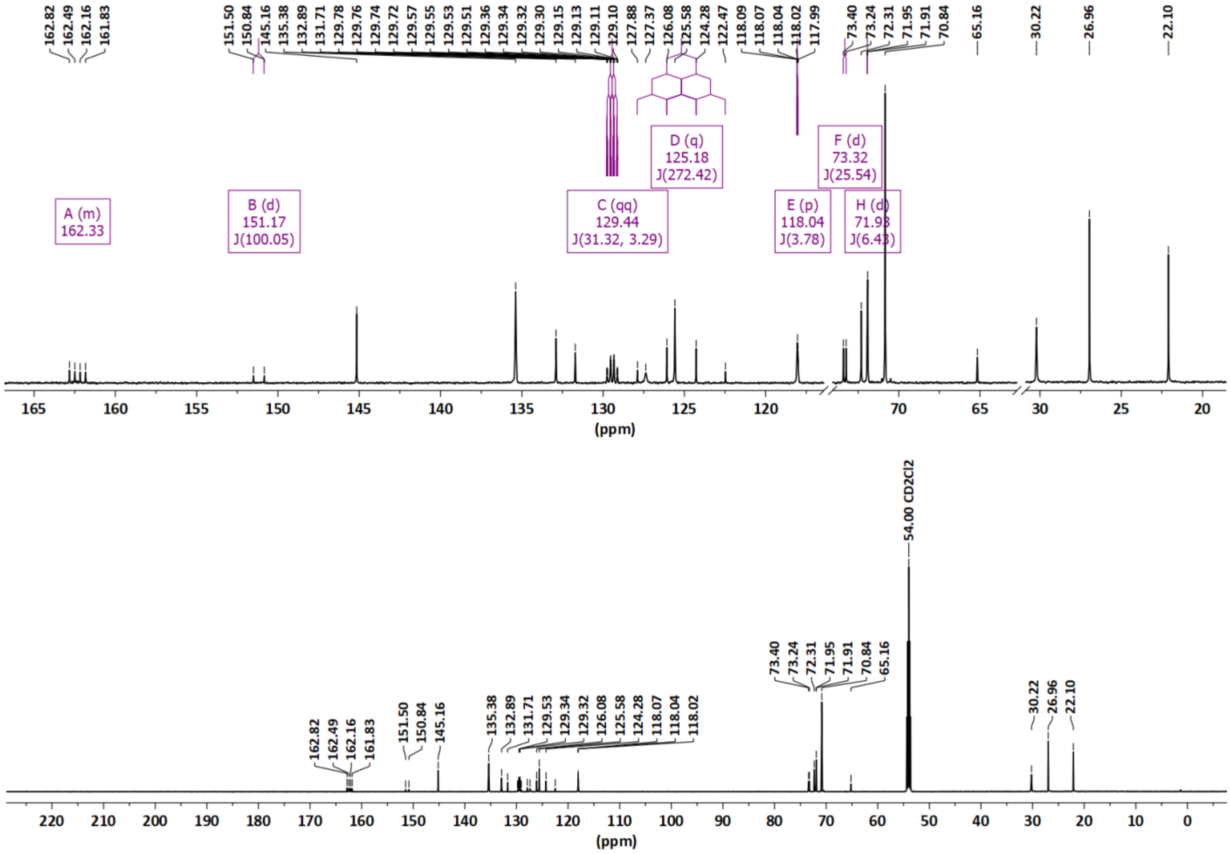

Figure S15. $^{13}C\{^{1}H\}$ NMR (CD_2Cl_2, 151 MHz) spectrum of **4**.

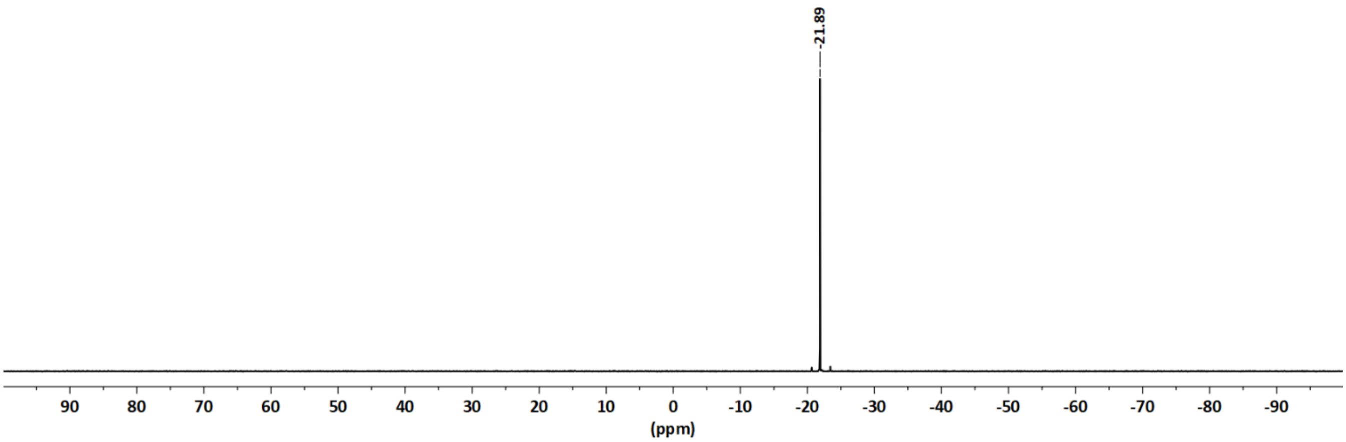

Figure S16. $^{31}P\{^{1}H\}$ NMR (CD_2Cl_2, 243 MHz) spectrum of **4**.

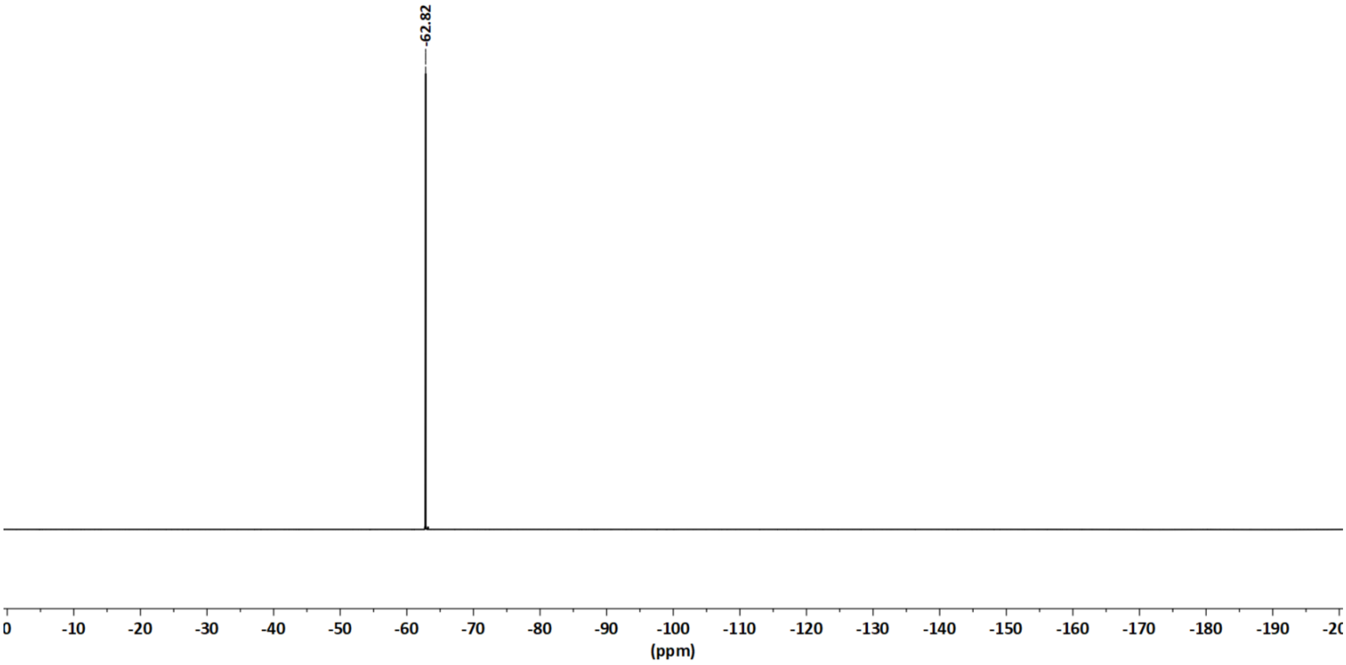

Figure S17. ^{19}F NMR (CD_2Cl_2, 565 MHz) spectrum of **4**.

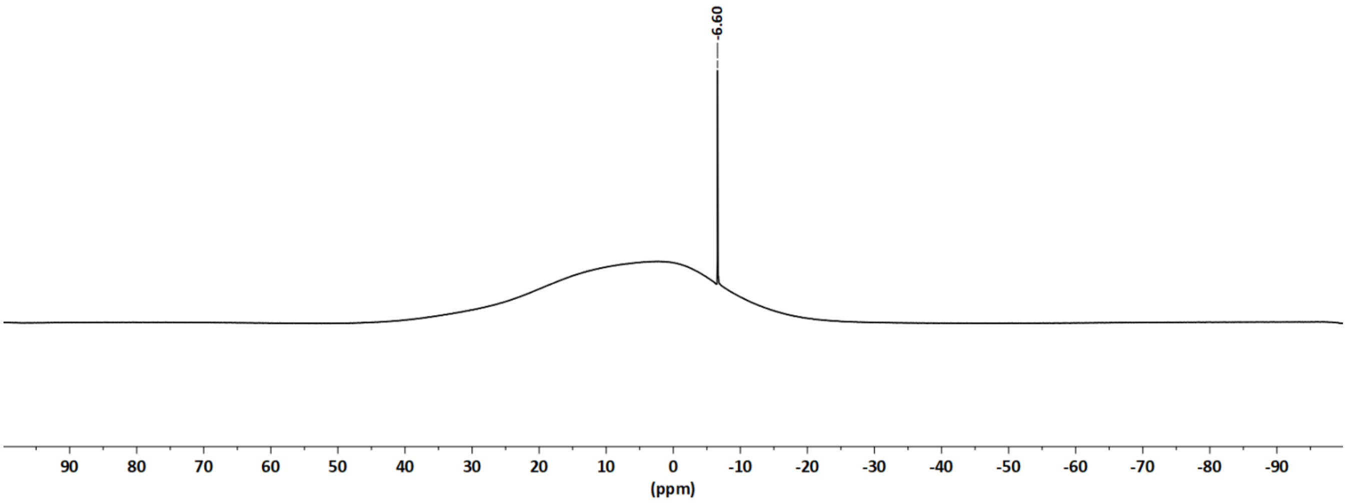

Figure S18. $^{11}B\{^{1}H\}$ NMR (CD_2Cl_2, 193 MHz) spectrum of **4**.

X-Ray diffraction studies

Intensity data of **1**–**4** was collected on a Bruker Venture D8 diffractometer at 100 K with graphite-monochromated Mo-Kα (0.7107 Å) radiation. All structures were solved by direct methods and refined based on F^2 by use of the SHELX program package as implemented in WinGX.[9,10] All non-hydrogen atoms were refined using anisotropic displacement parameters. Hydrogen atoms attached to carbon atoms were located form the difference Fourier map and refined freely for 1, 3 and 4. For 2, H atoms were treated as a mixture of freely refined and geometrically constrained positions within the ridding model. Crystal and refinement data are collected in Tables S1 and S2. Figures were created using DIAMOND.[11] Crystallographic data for the structural analyses have been deposited with the Cambridge Crystallographic Data Centre. Copies of this information may be obtained free of charge from The Director, CCDC, 12 Union Road, Cambridge CB2 1EZ, UK (Fax: +44-1223-336033; e-mail: deposit@ccdc.cam.ac.uk or http://www.ccdc.cam.ac.uk).

Table S1. Crystal data and structure refinement of **1** and **2**.

	**1**	**2**
Formula	$C_{20}H_{18}ClFe_2P$	$[C_{20}H_{18}Fe_2P][C_{32}H_{12}BF_{24}]$
Formula weight, g mol^{-1}	436.46	1264.24
Crystal system	Monoclinic	Monoclinic
Crystal size, mm	0.3 × 0.3 × 0.1	0.4 × 0.4 × 0.3
Space group	$P2_1/c$	$I1_2/c$
a, Å	9.7213(10)	19.4077(11)
b, Å	11.7026(11)	9.4833(4)
c, Å	15.0381(15)	26.4007(16)
α, º	90	90
β, º	91.298 (4)	93.297(4)
γ, º	90	90
V, Å^3	1710.4(3)	4851.0(5)
Z	4	4
ρ_{calcd}, g cm^{-3}	1.695	1.731
μ (Mo $K\alpha$), mm^{-1}	1.94	0.76
F(000)	888	2520
θ range, deg	2.73–30.55	2.39–30.55
Index ranges	$-13 \le k \le 13$	$-27 \le h \le 27$
	$-16 \le k \le 16$	$-13 \le k \le 12$
	$-21 \le l \le 21$	$-37 \le l \le 37$
No. of reflns collected	57386	134211
Completeness to θ_{max}	0.997	0.998
No. indep. Reflns	5245	7451
No. obsd reflns with ($I>2\sigma(I)$)	5245	6232
No. refined params	289	630
GooF (F^2)	1.041	1.034
R_1 (F) ($I > 2\sigma(I)$)	0.028	0.040
wR_2 (F^2) (all data)	0.074	0.100
Largest diff peak/hole, e Å^{-3}	1.08, −0.77	0.65, −0.88
CCDC number	1957841	1957842

Table S2. Crystal data and structure refinement of **3** and **4**.

	**3**	**4**
Formula	$[C_{38}H_{33}Fe_2P_2][C_{32}H_{12}BF_{24}]$	$[C_{47}H_{54}Fe_2N_2P][C_{32}H_{12}BF_{24}]$
Formula weight, g mol^{-1}	1526.51	1652.81
Crystal system	Triclinic	Monoclinic
Crystal size, mm	0.50 × 0.40 × 0.20	0.08 × 0.08 × 0.08
Space group	$P\bar{1}$	$P2_1/n$
a, Å	12.4192(5)	12.5994(9)
b, Å	14.1410(5)	15.7675(10)
c, Å	19.1133(7)	37.831(3)
α, º	97.3500(10)	90
β, º	105.5210(10)	91.789(2)
γ, º	92.4510(10)	90
V, Å^3	3197.3(2)	7511.9(9)
Z	2	4
ρ_{calcd}, g cm^{-3}	1.586	1.461
μ (Mo $K\alpha$), mm^{-1}	0.62	0.51
F(000)	1536	3368
θ range, deg	2.30–31.53	2.31–28.29
Index ranges	$-18 \leq h \leq 18$	$-21 \leq h \leq 21$
	$-20 \leq k \leq 20$	$-16 \leq k \leq 16$
	$-28 \leq l \leq 28$	$-49 \leq l \leq 50$
No. of reflns collected	104585	129087
Completeness to θ_{max}	0.995	0.998
No. indep. Reflns	21369	18772
No. obsd reflns with ($I>2\sigma(I)$)	18214	15823
No. refined params	1137	1285
GooF (F^2)	1.018	0.946
R_1 (F) ($I > 2\sigma(I)$)	0.036	0.049
wR_2 (F^2) (all data)	0.095	0.106
Largest diff peak/hole, e Å^{-3}	1.05, −0.69	0.48, −0.51
CCDC number	1957843	1957844

Computational data

Starting from the solid-state molecular geometries of **1**–**4** structural optimizations were conducted by density functional theory (DFT) at the B3PW91/6-311+G(2df,p)[12,13] level of theory using Gaussian09.[14] For Fe a fully relativistic effective core potential (ECP10MDF) and a corresponding cc-pVTZ basis set was utilized.[15,16] Normal mode (or frequency) analysis proved that all stationary points were at least local minima. The wavefunction files were employed for a topological analysis of the electron density according to the Atoms-In-Molecules (AIM) space-partitioning scheme using AIM2000,[17] whereas DGRID[18] was used to generate and analyze the Electron-Localizability-Indicator (ELI-D) related real-space bonding descriptors applying a grid step size of 0.05 a.u.. For ELI-D figures, additional grids of 0.15 a.u. step size were computed. NCI grids were generated with NCIplot.[19] Molecular orbitals (MO) were extracted from the formatted checkpoint files with the cubegen subroutine of Gaussian09. Natural bond orbitals (NBO) were calculated with NBO 5.9.[20] Bond paths are displayed with AIM2000, ELI-D and NCI figures are displayed with MolIso,[21] MO and NBO images are generated with GaussView 5.[22] The AIM topology parameters are collected in Table S3, whereas AIM charges (Q(AIM) are listed in Table S4. The P–C, P–N, P–P, and P–Cl bonds show both strong non-covalent and covalent bonding aspects, the contributions of which commonly are interpreted by means of the kinetic and total energy density over ED ratios, G/ρ or H/ρ. High positive G/ρ values suggest strong non-covalent contributions and high negative H/ρ values suggest strong covalent contributions, which is not mutually exclusive but could both be present. For the shorter P–C bonds (<1.9 Å) both aspects are higher than for the longer P–P bonds (>2.2 Å). ELI-D related parameters are compiled in Tables S5 and S6. The Raub Janzen indices of the donor acceptor bonds of **3** and **4** are 66.9 (P–P) and 87.5 (P–C), respectively.

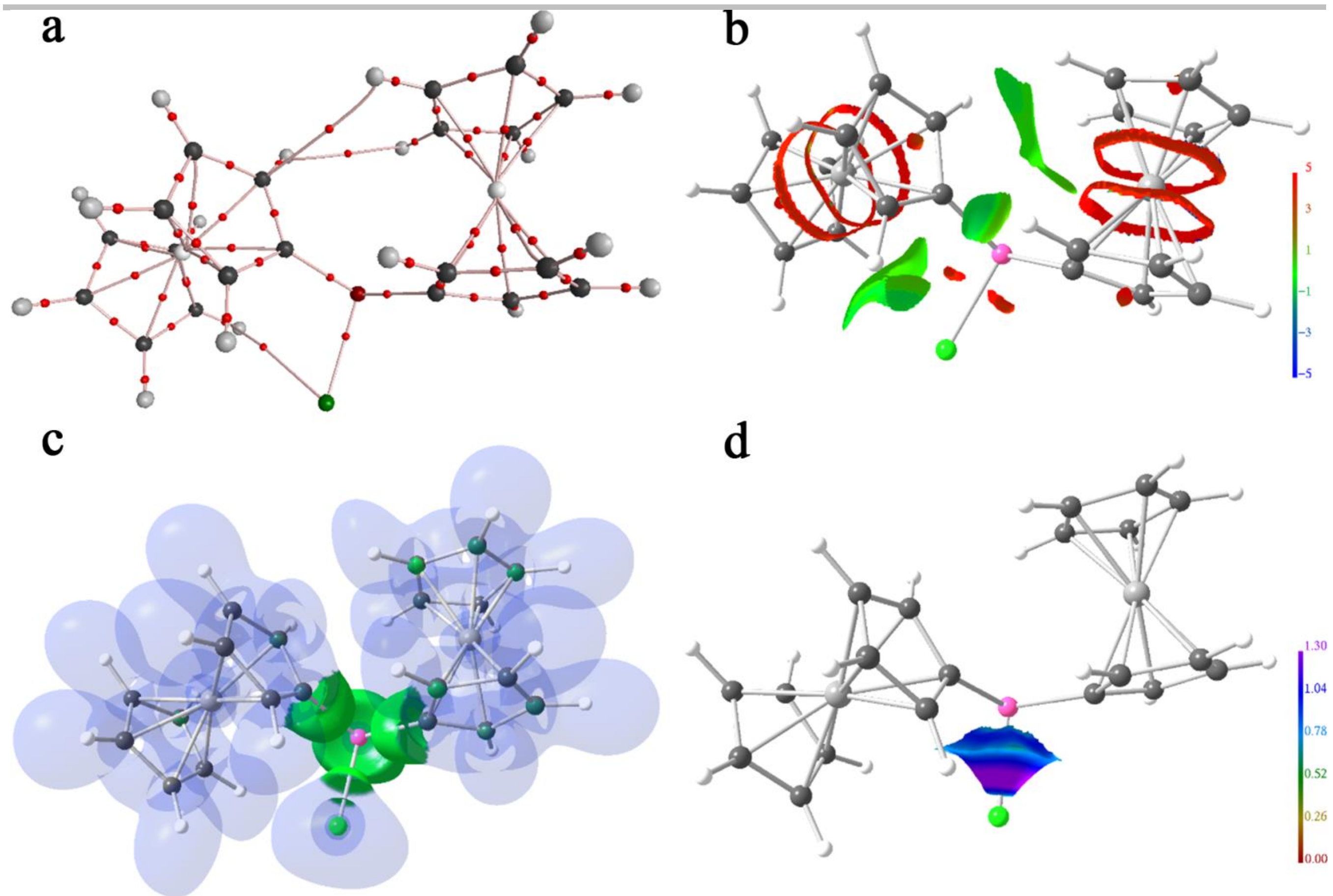

Figure S19. Real space bond indicator (RSBI) analysis of Fc$_2$PCl (**1**). (a) AIM bond topological analysis (b) NCI basins indicating non-covalent interactions (d) ELI-D indicating regions of electron localizability (e) ELI-D distribution mapped P–Cl bonding basin.

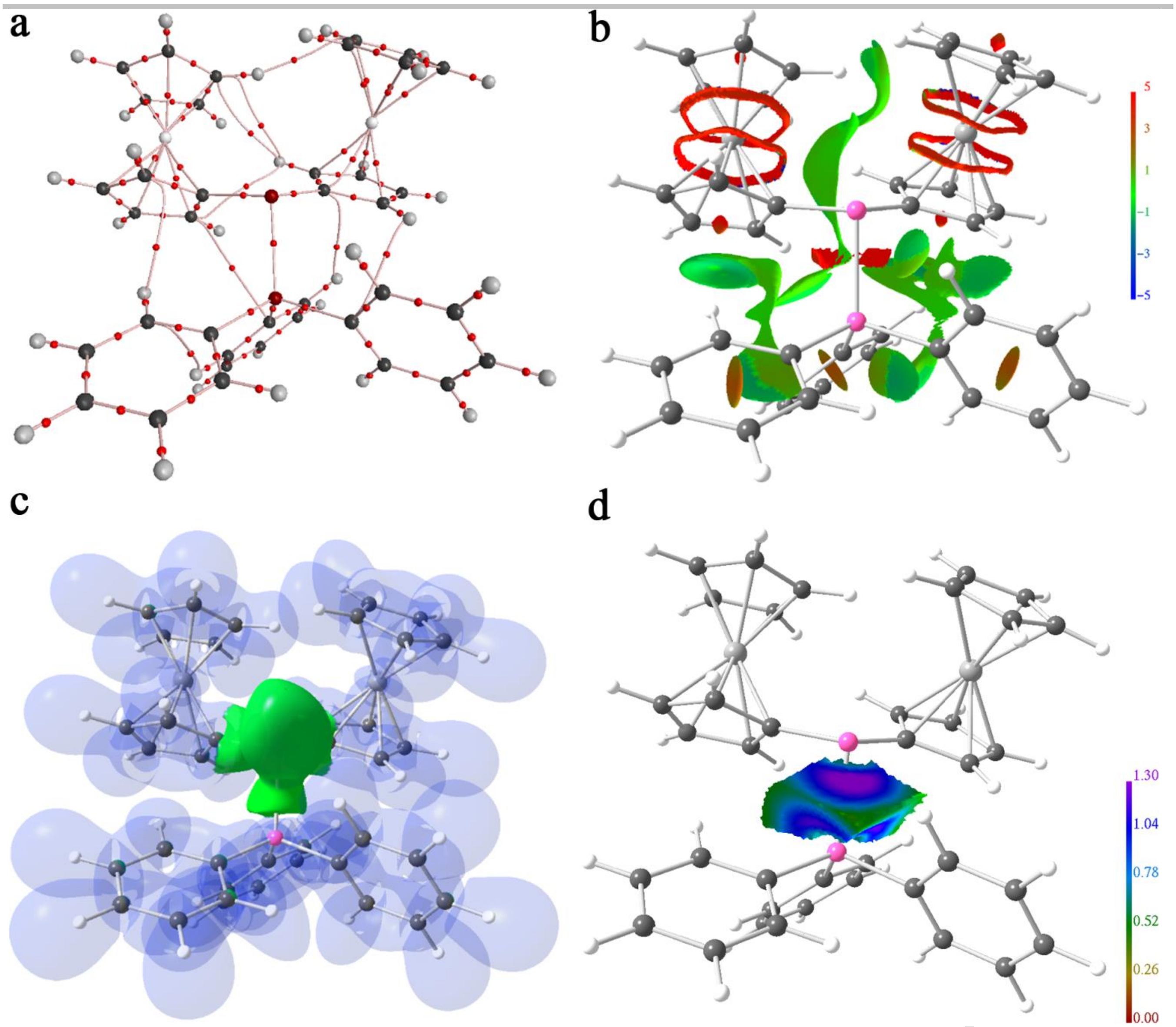

Figure S20. Real space bond indicator (RSBI) analysis of $[Fc_2P(PPh_3)][BAr^F{}_4]$ (**3**). (a) AIM bond topological analysis (b) NCI basins indicating non-covalent interactions (d) ELI-D indicating regions of electron localizability (e) ELI-D distribution mapped P–P bonding basin.

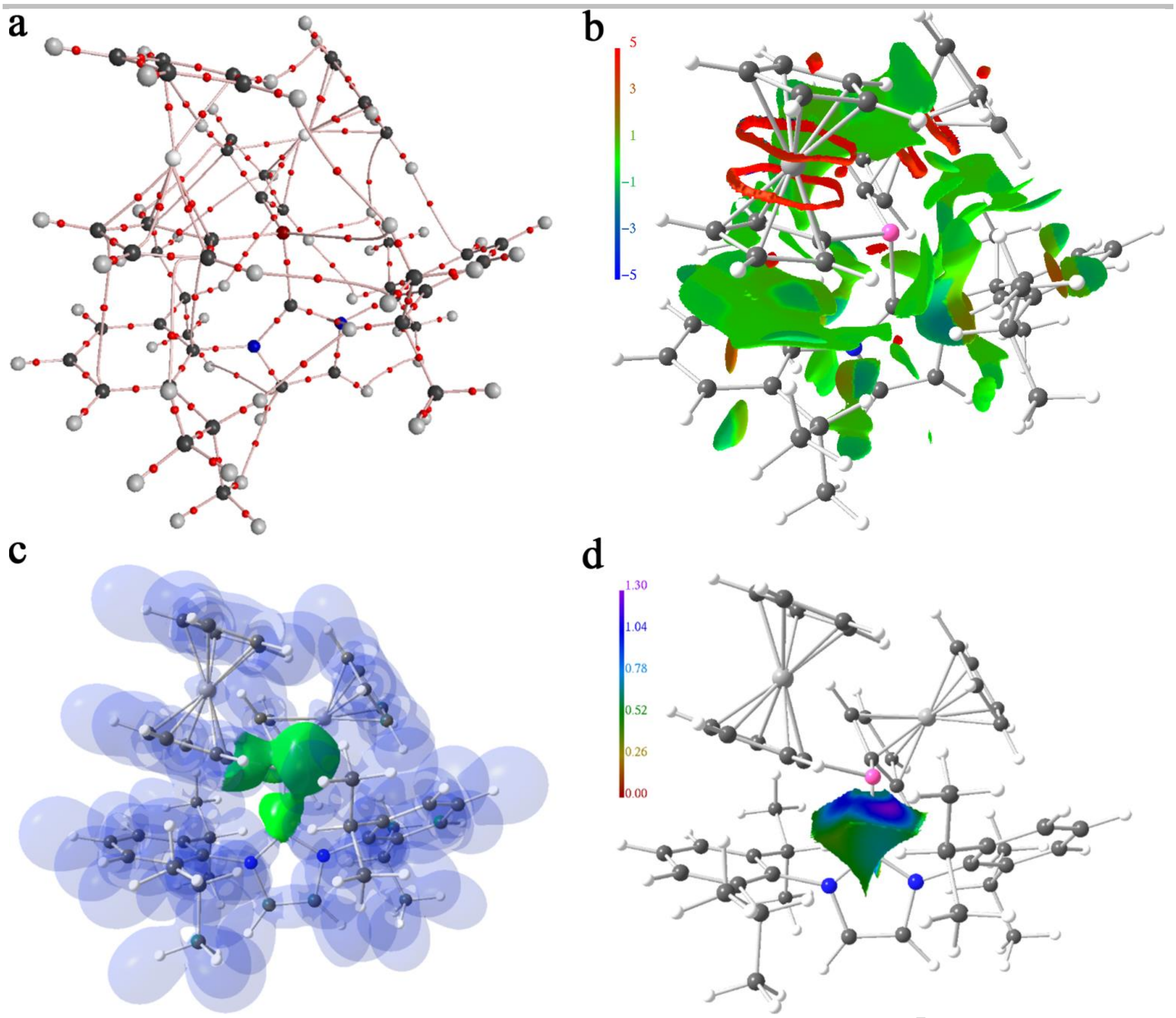

Figure S21. Real space bond indicator (RSBI) analysis of [Fc$_2$P(IPr)][BAr^F$_4$] (**4**). (a) AIM bond topological analysis (b) NCI basins indicating non-covalent interactions (d) ELI-D indicating regions of electron localizability (e) ELI-D distribution mapped P–P bonding basin.

Table S3. AIM-topology of **1 - 4**

		d [Å]	d_1/d [Å]	ε	ρ(r) [eÅ^{-3}]	∇^2ρ(r) [eÅ^{-5}]	G/ρ(r) [a.u.]	H/ρ(r) [a.u.]	d(P-Fe) [Å]
Fc_2PCl (**1**)	P–C	1.804	0.40	0.12	1.14	−5.6	0.67	−1.01	3.400
	P–C	1.799	0.40	0.07	1.15	−6.0	0.64	−1.01	3.317
	P–Cl	2.110	0.40	0.12	0.78	−3.0	0.44	−0.72	
$[Fc_2P]^+$ (**2**)	P–C	1.753	0.40	0.16	1.19	−3.0	0.85	−1.02	2.788
	P–C	1.753	0.40	0.15	1.19	−3.1	0.84	−1.02	2.780
$[Fc_2P(PPh_3)]^+$ (**3**)	P–C	1.801	0.40	0.15	1.13	−5.4	0.67	−1.00	3.402
	P–C	1.797	0.40	0.07	1.13	−5.2	0.68	−1.00	3.523
	P–P	2.236	0.46	0.12	0.77	−3.3	0.21	−0.51	
$[Fc_2P(IPr)]^+$ (**4**)	P–C	1.792	0.40	0.11	1.15	−5.2	0.70	−1.01	3.311
	P–C	1.811	0.40	0.19	1.12	−6.0	0.63	−1.00	3.340
	P–C	1.856	0.39	0.19	0.98	−2.6	0.73	−0.92	

For all bonds, $\rho(\mathbf{r})_{bcp}$ is the electron density at the bond critical point, $\nabla^2\rho(\mathbf{r})_{bcp}$ is the corresponding Laplacian, ε is the bond ellipticity, $G/\rho(\mathbf{r})_{bcp}$ and $H/\rho(\mathbf{r})_{bcp}$ are the kinetic and total energy density over $\rho(\mathbf{r})_{bcp}$ ratios.

Table S4. Q(AIM) charges of **1 - 4**

$[Fc_2P]^+$ (**2**)		Fc_2PCl (**1**)		$[Fc_2P(PPh_3)]^+$ (**3**)		$[Fc_2P(IPr)]^+$ (**4**)	
Fc1	−0.20	Fc1	−0.50	Fc1	−0.44	Fc1	−0.44
Fc2	−0.21	Fc2	−0.52	Fc2	−0.43	Fc2	−0.46
P	1.42	P	1.59	P	1.26	P	1.67
		Cl	−0.54	PPh_3	0.65	IPr	0.27
Σ	1.01	Σ	0.02	Σ	1.04	Σ	1.04

Table S5. ELI parameters of Fc_2PCl (**1**, left) and $[Fc_2P]^+$ (**2**, right).

basin	V_{ELI} [Å^3]	N_{ELI} [e]	γ_{ELI}	basin	V_{ELI} [Å^3]	N_{ELI} [e]	γ_{ELI}
C–P1	4.9	2.23	1.90	C–P	7.7	2.74	1.84
C–P1	4.9	2.25	1.90	C–P	7.7	2.74	1.84
Fe1–C	0.5	2.15	1.79	Fe1–C	0.5	2.15	1.75
Fe1–C	0.5	2.16	1.80	Fe1–C	0.5	2.16	1.76
Fe2–C	0.5	2.15	1.79	Fe2–C	0.5	2.16	1.75
Fe2–C	0.5	2.16	1.79	Fe2–C	0.5	2.16	1.76
LP–Fe1	0.9	2.18	1.54	LP–Fe1	0.8	2.29	1.56
LP–Fe1	0.9	2.20	1.54	LP–Fe1	0.8	2.32	1.56
LP–Fe1	1.0	2.40	1.53	LP–Fe1	1.0	2.36	1.55
LP–Fe1	1.0	2.55	1.53	LP–Fe1	1.1	2.32	1.55
LP–Fe2	0.9	2.15	1.54	LP–Fe2	0.8	2.30	1.56
LP–Fe2	0.9	2.22	1.54	LP–Fe2	0.8	2.32	1.56
LP–Fe2	1.0	2.40	1.54	LP–Fe2	1.0	2.36	1.55
LP–Fe2	1.0	2.56	1.53	LP–Fe2	1.1	2.32	1.55
C–Fe1	1.1	0.36	1.32	C–Fe1	1.1	0.33	1.32
C–Fe1	1.0	0.29	1.31	C–Fe1	1.1	0.30	1.31
C–Fe1	0.8	0.24	1.31	C–Fe1	1.1	0.27	1.31
C–Fe1	0.9	0.24	1.31	C–Fe1	0.6	0.18	1.29
C–Fe1	1.1	0.32	1.32	C–Fe1	0.5	0.17	1.29
C–Fe1	0.8	0.23	1.30	C–Fe1	1.1	0.27	1.31
C–Fe1	0.9	0.25	1.31				
C–Fe1	1.0	0.26	1.31	C–Fe2	1.1	0.33	1.32
C–Fe1	1.0	0.27	1.31	C–Fe2	1.1	0.30	1.31
C–Fe1	1.0	0.26	1.31	C–Fe2	1.1	0.27	1.31
				C–Fe2	0.6	0.18	1.29
C–Fe2	1.0	0.32	1.31	C–Fe2	0.5	0.17	1.29
C–Fe2	1.0	0.28	1.31	C–Fe2	1.1	0.27	1.31
C–Fe2	0.8	0.22	1.31				
C–Fe2	0.8	0.22	1.31				
C–Fe2	1.1	0.30	1.31				
C–Fe2	0.9	0.25	1.31				
C–Fe2	0.9	0.26	1.31				
C–Fe2	0.9	0.25	1.31				
C–Fe2	1.0	0.27	1.31				
C–Fe2	0.9	0.26	1.31				
LP–P	16.6	2.13	2.84	LP–P	18.2	2.66	2.56
Cl–P	1.9	1.16	1.47				
LP–Cl	11.3	2.35	1.69				
LP–Cl	20.5	4.24	1.69				

N_{ELI} and V_{ELI} are electron populations and volumes of related ELI-D basins, γ_{ELI} is the ELI-D value at the attractor position.

Table S6. ELI parameters of $[Fc_2P(PPh_3)]^+$ (**3**, left) and $[Fc_2P(IPr)]^+$ (**4**, right).

basin	V_{ELI} [Å^3]	N_{ELI} [e]	γ_{ELI}	basin	V_{ELI} [Å^3]	N_{ELI} [e]	γ_{ELI}
C–P1	5.1	2.22	1.87	C–P	5.3	2.25	1.88
C–P1	5.3	2.22	1.86	C–P	5.1	2.18	1.89
Fe1–C	0.5	2.15	1.80	Fe1–C	0.5	2.15	1.79
Fe1–C	0.5	2.15	1.80	Fe1–C	0.5	2.16	1.80
Fe2–C	0.5	2.15	1.79	Fe2–C	0.5	2.16	1.80
Fe2–C	0.5	2.16	1.80	Fe2–C	0.5	2.16	1.80
LP–Fe1	0.8	2.30	1.54	LP–Fe1	0.8	2.16	1.54
LP–Fe1	0.9	2.39	1.54	LP–Fe1	0.9	2.26	1.54
LP–Fe1	1.0	2.27	1.54	LP–Fe1	0.9	2.55	1.54
LP–Fe1	1.2	2.40	1.54	LP–Fe1	1.0	2.35	1.54
				LP–Fe2	0.4	0.90	1.53
LP–Fe2	0.8	2.25	1.54	LP–Fe2	0.8	2.00	1.53
LP–Fe2	0.9	2.24	1.54	LP–Fe2	0.8	2.07	1.53
LP–Fe2	0.9	2.34	1.54	LP–Fe2	0.9	2.18	1.54
LP–Fe2	1.0	2.49	1.54	LP–Fe2	0.9	2.20	1.54
C–Fe1	1.1	0.37	1.33	C–Fe1	1.0	0.34	1.32
C–Fe1	1.1	0.33	1.33	C–Fe1	1.2	0.34	1.32
C–Fe1	0.6	0.19	1.30	C–Fe1	0.8	0.24	1.31
C–Fe1	0.7	0.20	1.30	C–Fe1	0.7	0.20	1.30
C–Fe1	1.1	0.28	1.32	C–Fe1	1.1	0.30	1.32
C–Fe1	1.1	0.28	1.31	C–Fe1	1.1	0.28	1.31
C–Fe1	0.9	0.26	1.31	C–Fe1	1.0	0.26	1.31
C–Fe1	0.7	0.22	1.30	C–Fe1	0.8	0.23	1.30
C–Fe1	0.9	0.24	1.31	C–Fe1	0.9	0.25	1.31
C–Fe1	1.0	0.26	1.31	C–Fe1	1.1	0.28	1.31
C–Fe2	1.7	0.47	1.36	C–Fe2	0.7	0.25	1.31
C–Fe2	0.6	0.18	1.30	C–Fe2	1.1	0.32	1.32
C–Fe2	0.6	0.19	1.30	C–Fe2	0.9	0.25	1.31
C–Fe2	1.1	0.30	1.32	C–Fe2	0.9	0.25	1.31
C–Fe2	0.8	0.23	1.30	C–Fe2	1.2	0.34	1.33
C–Fe2	1.0	0.27	1.31	C–Fe2	1.0	0.26	1.31
C–Fe2	0.9	0.25	1.31	C–Fe2	0.9	0.25	1.31
C–Fe2	0.7	0.22	1.30	C–Fe2	0.9	0.24	1.31
C–Fe2	1.0	0.27	1.31	C–Fe2	0.9	0.26	1.31
				C–Fe2	1.0	0.26	1.31
LP–P1	16.5	2.09	2.61	LP–P	13.4	2.07	2.67
P2–P1	5.7	1.95	1.87	C–P	6.5	2.49	1.95
C–P2	5.4	2.25	1.91	LP–N	1.2	0.43	1.48
C–P2	5.6	2.27	1.90				
C–P2	5.5	2.25	1.90				

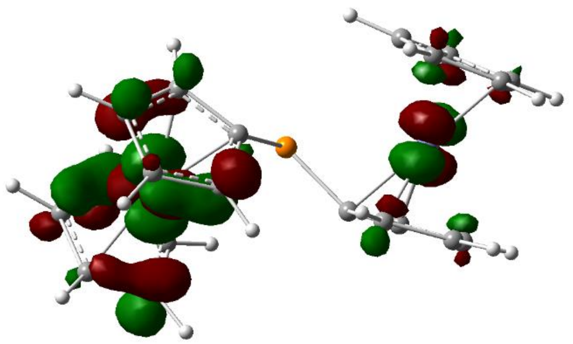

LUMO+4 (0.04 a.u.)

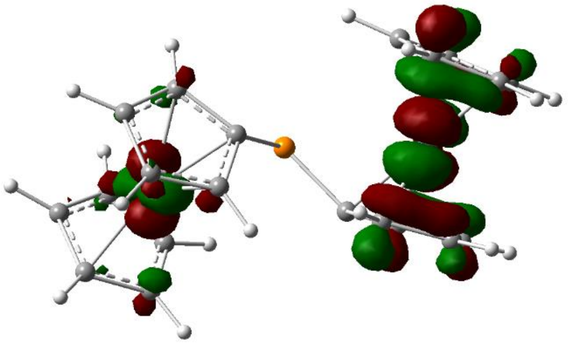

LUMO+3 (0.04 a.u.)

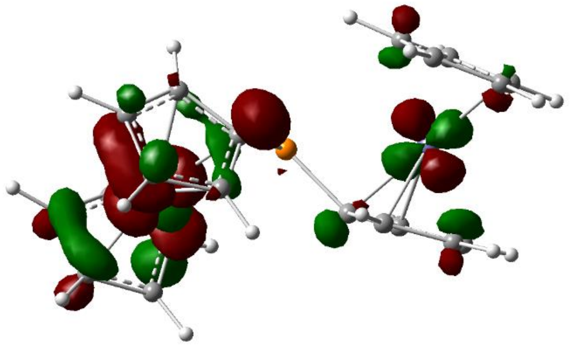

LUMO+2 (0.04 a.u.)

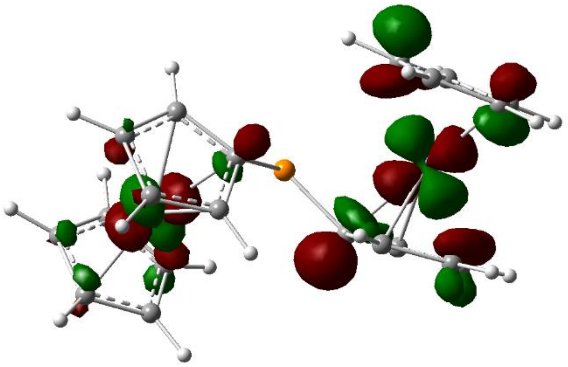

LUMO+1 (0.04 a.u.)

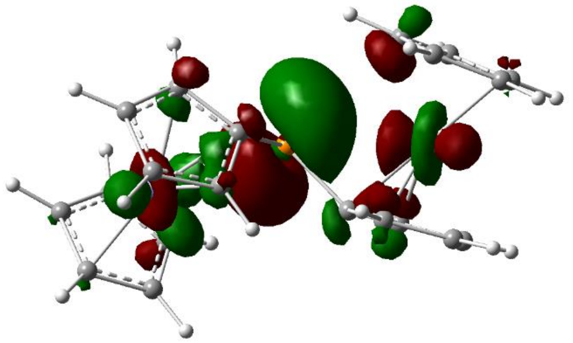

LUMO (0.04 a.u.)

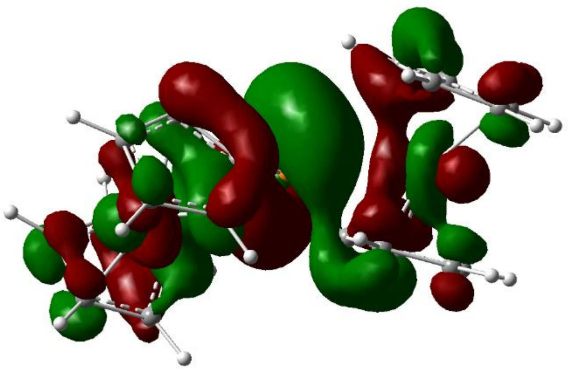

LUMO (0.02 a.u.)

Figure S22. Frontier orbitals of **2** (LUMO to LUMO+4).

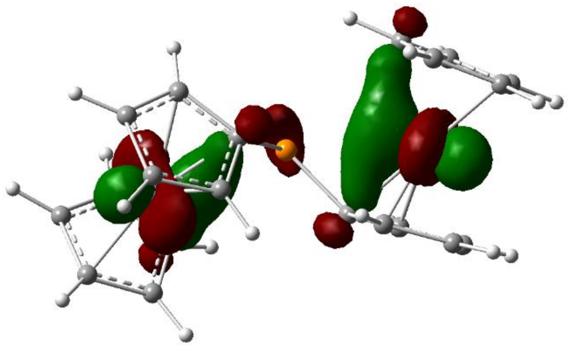

HOMO (0.04 a.u.)

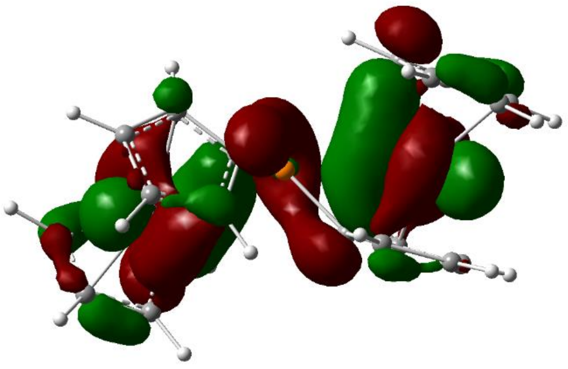

HOMO (0.02 a.u.)

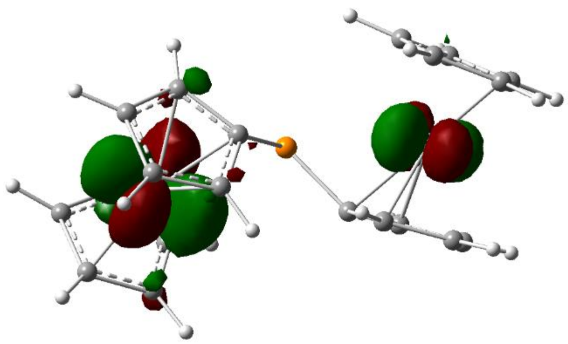

HOMO-1 (0.04 a.u.)

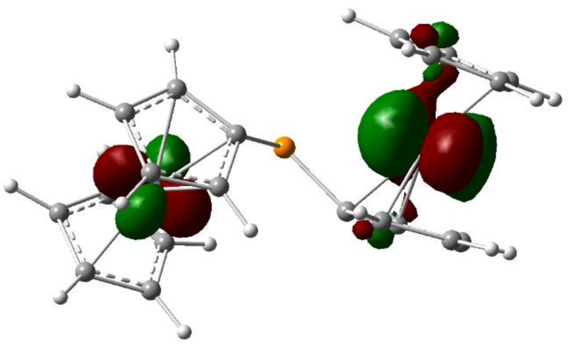

HOMO-2 (0.04 a.u.)

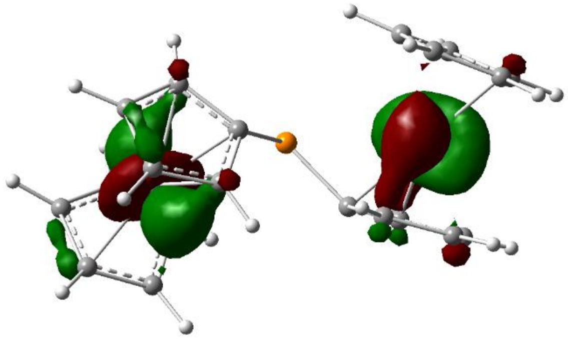

HOMO-3 (0.04 a.u.)

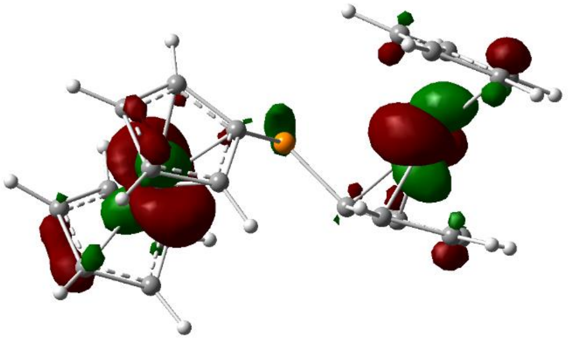

HOMO-4 (0.04 a.u.)

Figure S23. Frontier orbitals of **2** (HOMO to HOMO+4).

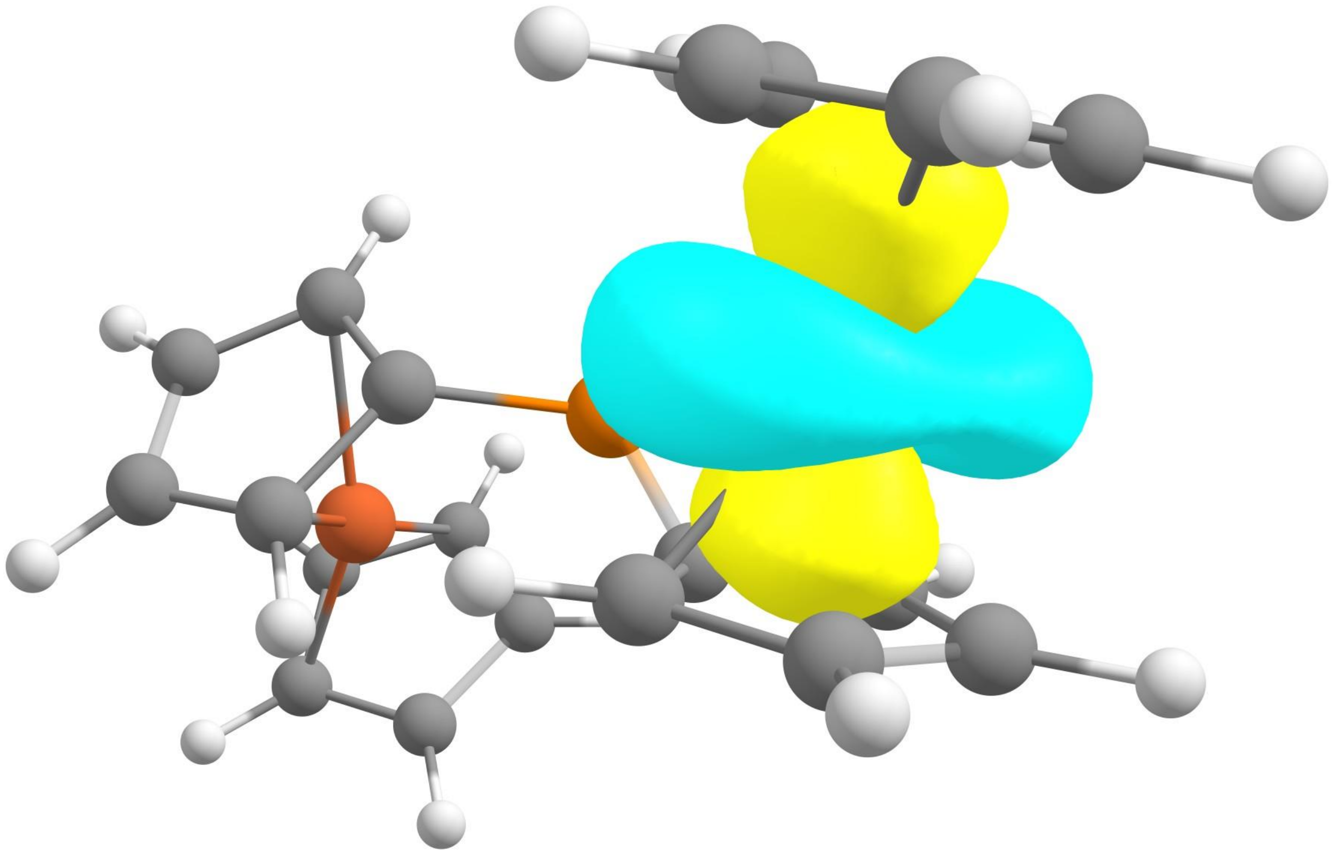

Figure S24. NBO of LP1(Fe1) of **2**, populated with 1.94e.

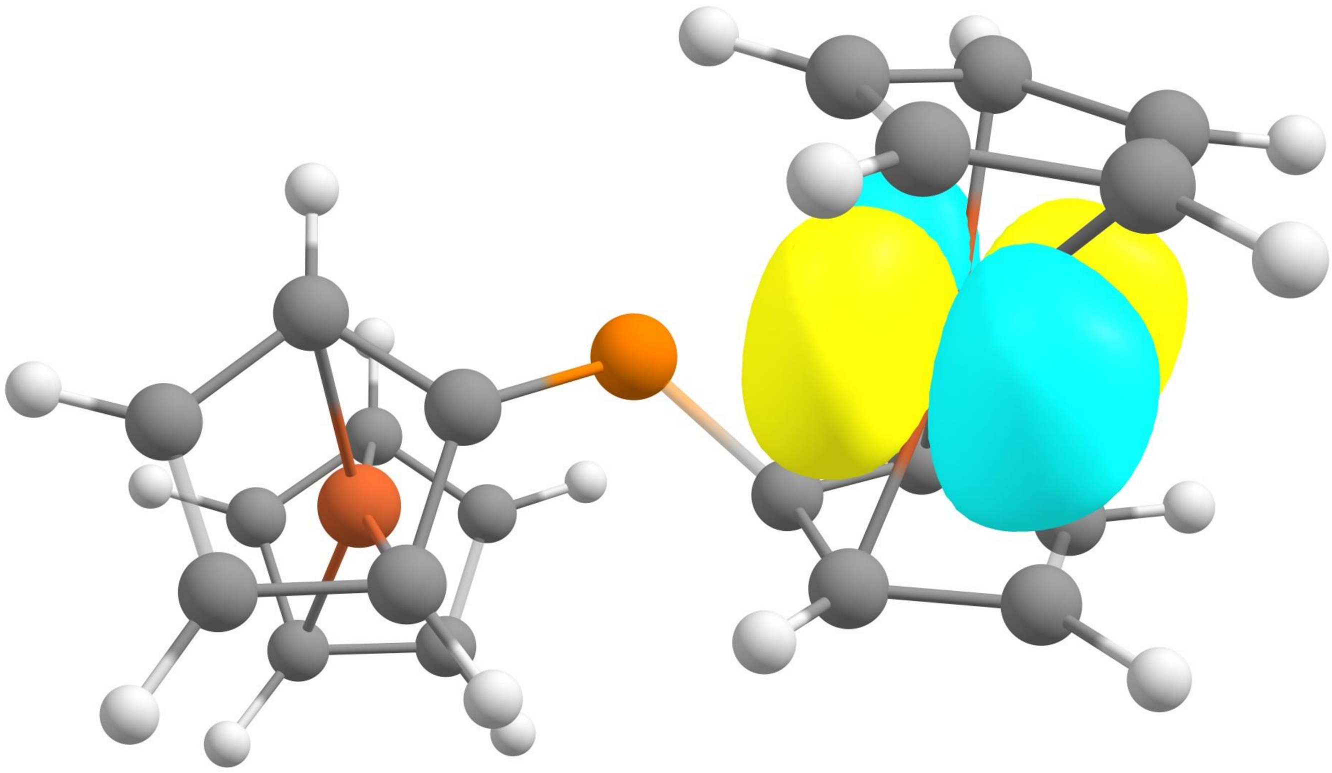

Figure S25. NBO of LP2(Fe1) of **2**, populated with 1.88e.

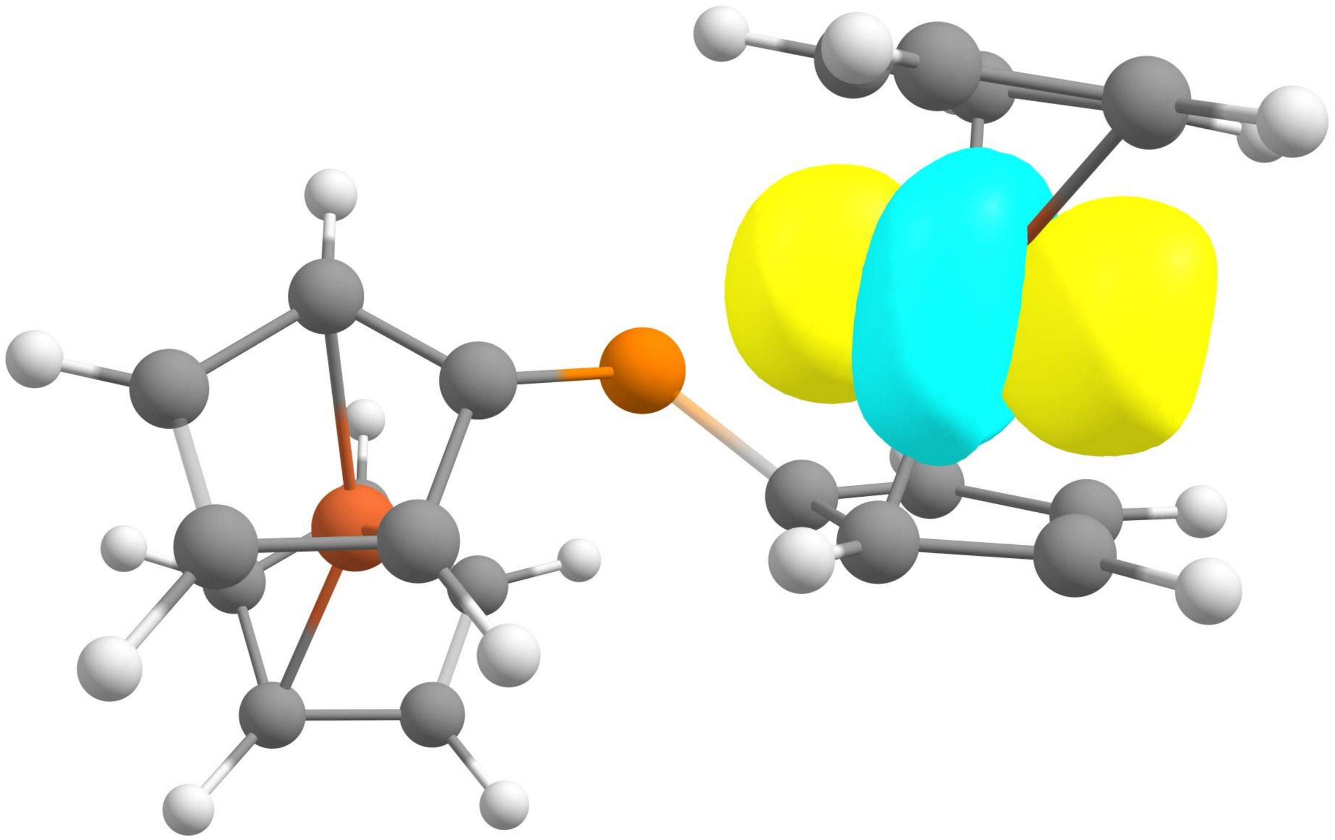

Figure S26. NBO of LP3(Fe1) of **2**, populated with 1.73e.

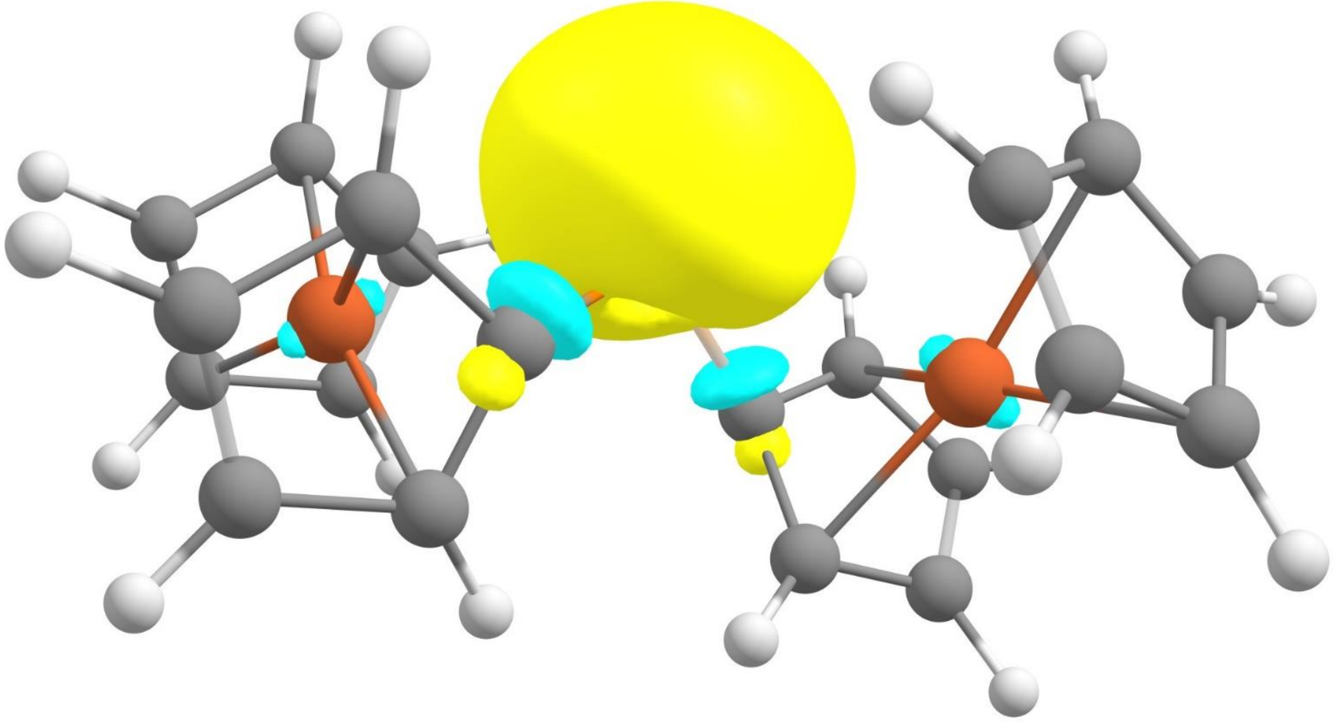

Figure S27. NBO of LP1(P) of **2**, populated with 1.95e.

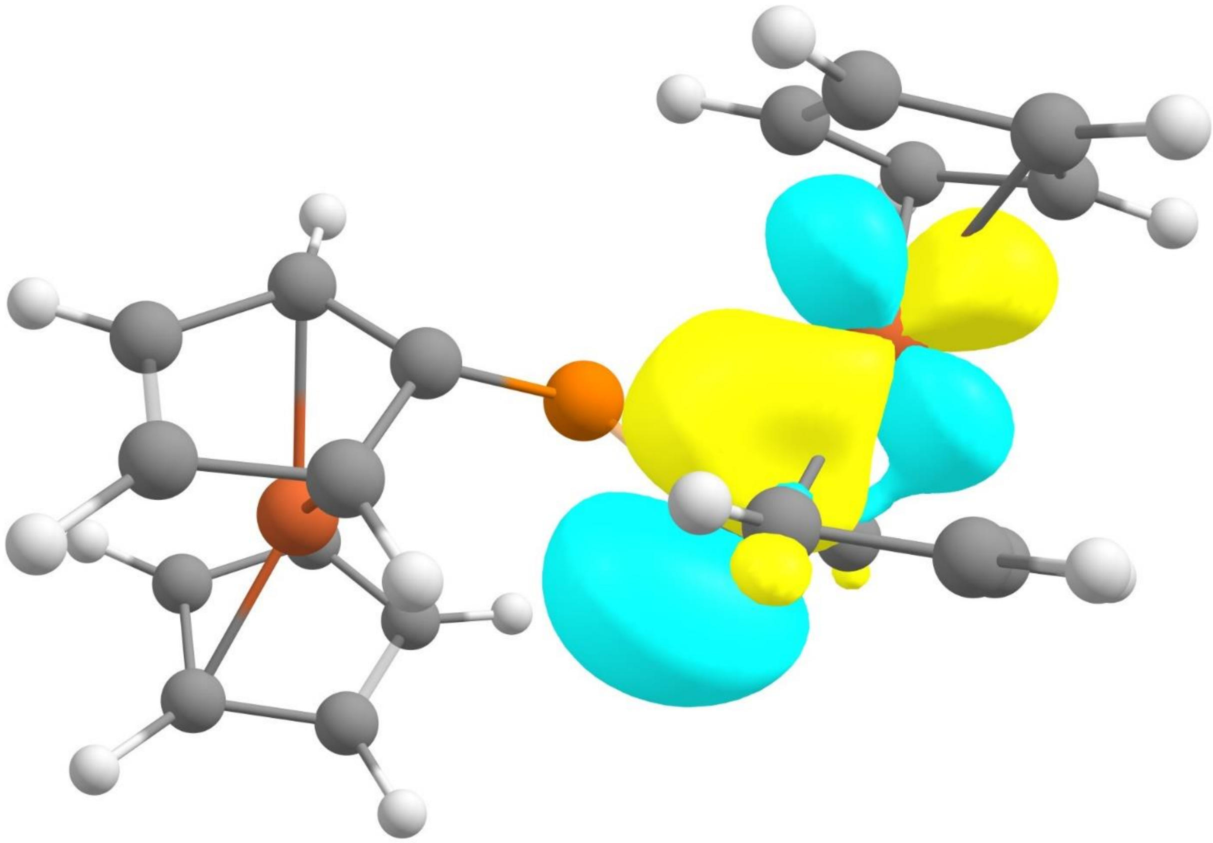

Figure S28. NBO of BD(Fe1-C2) of **2**, populated with 1.44e.

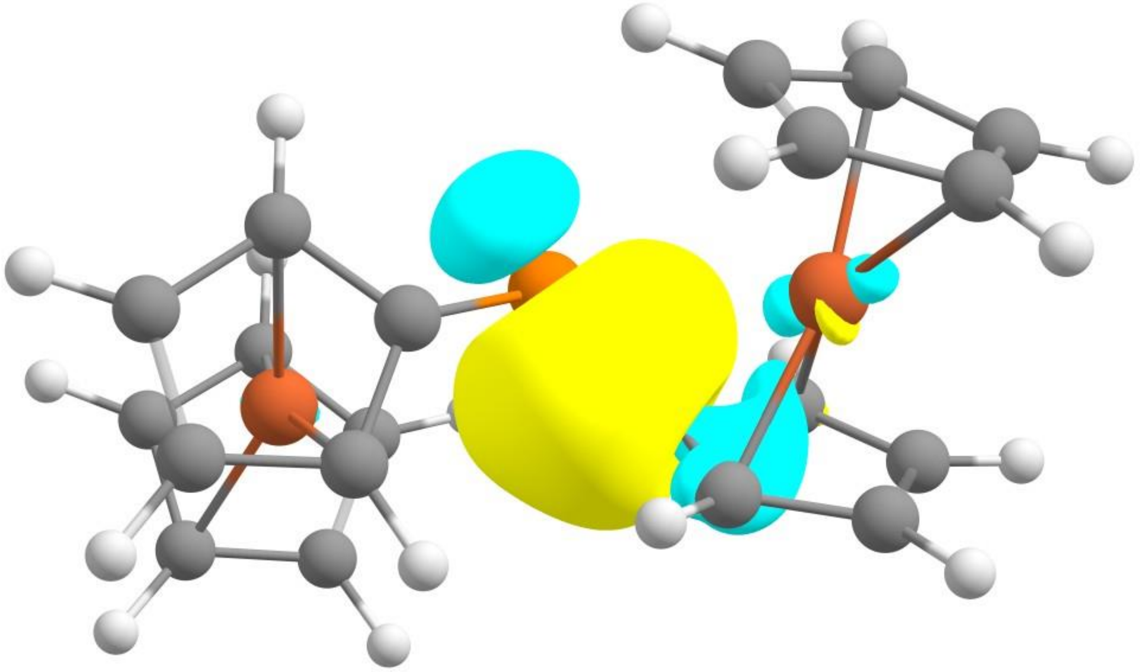

Figure S29. NBO of the P-C bond of **2**, populated with 1.96e.

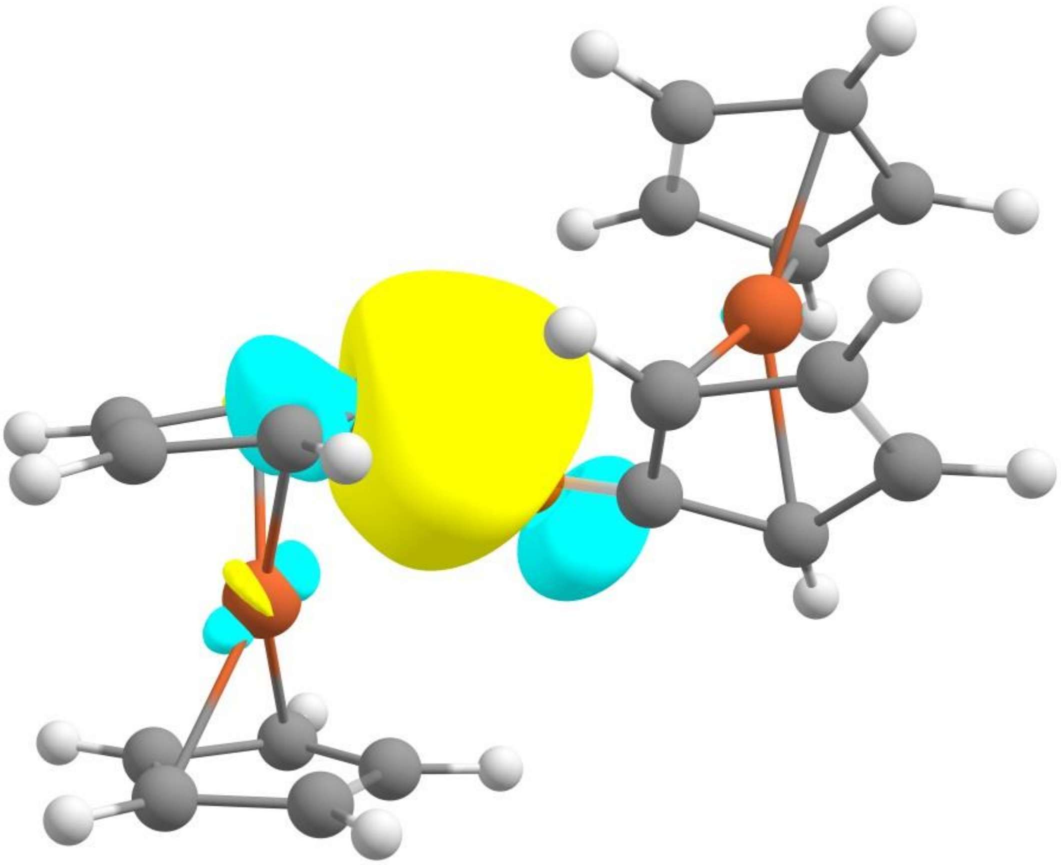

Figure S30. NBO of the P-C bond of **2**, populated with 1.96e.

References

1. D. Guillaneux, H. B. Kagan, *J. Org. Chem.* **1995**, *60*, 2502–2505.
2. C. Förster, K. Heinze, *Z. Anorg. Allg. Chem.* **2015***, 641*, 517–520.
3. N. A. Yakelis, R. G. Bergman, *Organometallics* **2005**, *24*, 3579–3581.
4. M. Brookhart, B. Grant, A. F. Volpe Jr., *Organometallics* **1992**, *11*, 3920–3922.
5. S. J. Conway, J. C. Miller, A. D. Bond, B. P. Clark, D. E. Jane, *J. Chem. Soc., Perkin Trans. 1,* **2002***,* 1625–1627 .
6. L. Hintermann, *Beilstein J. Org. Chem.* **2007***, 3. doi:10.1186/1860-5397-3-22*.
7. M. Pompeo R.D. J. Froese, N. Hadei, M. G. Organ, *Angew. Chem. Int. Ed.* **2012**, *51*, 11354–11357.
8. G. R. Fulmer, A. J. M. Miller, N. H. Sherden, H. E. Gottlieb, A. Nudelman, B. M. Stoltz, J. E. Bercaw, K. I. Goldberg, *Organometallics* **2010**, *29*, 2176–2179.
9. G. M. Sheldrick, *Acta Cryst*. **2008**, *A64*, 112–122.
10. L. Farrugia, *J. Appl. Cryst*. **1999**, *32*, 837–838.
11. K. Brandenburg, Diamond, version 4.0.4, Crystal Impact GbR: Bonn, Germany, **2012**.
12. J. P Perdew, J. A. Chevary, S. H. Vosko, K. A. Jackson, M. R. Pederson, D. J. Singh, C. Fiolhais, *Phys. Rev*. B **1992**, *46*, 6671–6687.
13. A. D. Becke, *J. Chem. Phys*. **1993**, *98*, 5648–5652.
14. M. J. Frisch, G. W. Trucks, H. B. Schlegel, G. E. Scuseria, M. A. Robb, J. R. Cheeseman, G. Scalmani, V. Barone, B. Mennucci, G. A. Petersson, H. Nakatsuji, M. Caricato, X. Li, H. P. Hratchian, A. F. Izmaylov, J. Bloino, G. Zheng, J. L. Sonnenberg, M. Hada, M. Ehara, K. Toyota, R. Fukuda, J. Hasegawa, M. Ishida, T. Nakajima, Y. Honda, O. Kitao, H. Nakai, T. Vreven, J. A. Montgomery, Jr., J. E. Peralta, F. Ogliaro, M. Bearpark, J. J. Heyd, E. Brothers, K. N. Kudin, V. N. Staroverov, R. Kobayashi, J. Normand, K. Raghavachari, A. Rendell, J. C. Burant, S. S. Iyengar, J. Tomasi, M. Cossi, N. Rega, J. M. Millam, M. Klene, J. E. Knox, J. B. Cross, V. Bakken, C. Adamo, J. Jaramillo, R. Gomperts, R. E. Stratmann, O. Yazyev, A. J. Austin, R. Cammi, C. Pomelli, J. W. Ochterski, R. L. Martin, K. Morokuma, V. G. Zakrzewski, G. A. Voth, P. Salvador, J. J. Dannenberg, S. Dapprich, A. D. Daniels, Ö. Farkas, J. B. Foresman, J. V. Ortiz, J. Cioslowski, D. J. Fox, Gaussian 09, Revision B.01, Gaussian Inc., Wallingford CT, **2010**.
15. M. Dolg, U. Wedig, H. Stoll, H. Preuss, *J. Chem. Phys*. **1987**, *86*, 866–872.
16. J.M.L. Martin, A. Sundermann, *J. Chem. Phys*. **2001**, *114*, 3408–3420.
17. F. Biegler-König, J. Schönbohm, D. Bayles, *J. Comput. Chem*. **2001**, *22*, 545–559.
18. M. Kohout, DGRID-4.6 Radebeul, **2015**.
19. J. Contreras-García, E. Johnson, S. Keinan, R. Chaudret, J.-P. Piquemal, D. Beratan, W. Yang, *J. Chem. Theor. Comp.* **2011**, *7*, 625–632.
20. E. D. Glendening, J. K. Badenhoop, A. E. Reed, J. E. Carpenter, J. A. Bohmann, C. M. Morales, F. Weinhold, NBO 5.9, Theoretical Chemistry Institute, University of Wisconsin, Madison, WI, 2009; http://www.chem.wisc.edu/~nbo5
21. C. B. Hübschle, P. Luger, *J. Appl. Crystallogr*. **2006**, *39*, 901–904.
22. Æ. Frisch, H. P. Hratchian, R. D. Dennington II, T. A. Keith, J. Millam, A. B. Nielsen, A. J. Holderand J. Hiscocks, GaussView 5, Gaussian Inc., Wallingford, CT, **2009**.
